# Influence of gut microbial metabolites on tumor immunotherapy: mechanisms and potential natural products

**DOI:** 10.3389/fimmu.2025.1552010

**Published:** 2025-02-24

**Authors:** Dongyang Li, Xintian Lan, Linyi Xu, Shuo Zhou, Haoming Luo, Xiaoying Zhang, Wenbo Yu, Yonggang Yang, Xiaoxue Fang

**Affiliations:** ^1^ Changchun University of Chinese Medicine, Changchun, China; ^2^ School of Pharmacy, Changchun University of Chinese Medicine, Changchun, China; ^3^ Department of Clinical Pharmacy, The First Hospital of Jilin University, Changchun, China

**Keywords:** gut microbial metabolites, tumor, natural products, immune, mechanism

## Abstract

In recent years, tumor immunotherapy has made significant breakthroughs in the treatment of malignant tumors. However, individual differences in efficacy have been observed in clinical practice. There is increasing evidence that gut microbial metabolites influence the efficacy of distal tumor immunotherapy via the gut-liver axis, the gut-brain axis and the gut-breast axis, a process that may involve modulating the expression of immune cells and cytokines in the tumor microenvironment (TME). In this review, we systematically explore the relationship between gut microbial metabolites and tumor immunotherapy, and examine the corresponding natural products and their mechanisms of action. The in-depth exploration of this research area will provide new ideas and strategies to enhance the efficacy of tumor immunotherapy and mitigate adverse effects.

## 1 Introduction

Tumor immunotherapy has recently emerged as a breakthrough approach to the treatment of malignant tumors, and unlike traditional cancer treatments such as radiotherapy and chemotherapy, immunotherapy harnesses the host's immune system to target and eliminate cancer cells (1). In recent decades, with the continuous progress of our understanding of the cancer immunosuppressive microenvironment, immunotherapy has become the key pillar of cancer treatment, including immunosuppressive, chimeric antigen receptor (CAR) therapy, cancer vaccination and oncolytic virus therapy (1). However, long-term chronic stimulation of immune cells by tumor antigens and the uncontrolled inflammation associated with carcinogenesis ultimately impairs anti-tumor immunity and promotes tumor progression (2), resulting in a situation where not all patients benefit and some may even suffer serious immune-related adverse events (3).

The gut flora is the most important metabolic organ in the human body and consists of a wide range of microorganisms, including bacteria, archaea, viruses, unicellular eukaryotes and fungi (4). Numerous studies have shown that gut microbes influence cancer development through a variety of mechanisms, in which bacterial metabolites may play an important role (5, 6). Bacterial metabolites such as short-chain fatty acids (SCFAs), bile acids, lactic acid, spermidine, indole and retinoic acid have been shown to link the gut microbiota to systemic immunity, which in turn influences cancer development (7). For example, microbial metabolites have been shown to influence the initiation and progression of endocrine tumors by altering various signaling pathways (8–10). Acetic acid improves pancreatitis and its sequelae and may reduce the incidence of pancreatic cancer (11). Lactate-mediated epigenetic reprogramming regulates the formation of human pancreatic cancer-associated fibroblasts and the ultrastructural differentiation of sodium butyrate-treated human pancreatic cancer cell lines, alterations that have the potential to increase the invasiveness of pancreatic cancer cells (12, 13). It has also been shown that butyrate has an inhibitory effect on the proliferation of pancreatic cancer cells by inducing a specific secretory phenotype through structural changes (14). A study of patients with pancreatic cancer showed that all diagnosed patients had elevated bile acid levels (15). Other studies have shown that patients with ovarian cancer have reduced blood levels of tryptophan and indolepropionic acid, a trend that becomes more pronounced as the disease progresses (16–19).

In recent years, microbial metabolites have been shown to play an important role in tumor immunotherapy by activating the immune system to eliminate tumor cells and prevent the development of drug resistance (20). Therefore, in-depth exploration of the crosstalk between gut microbial metabolites and tumor immunotherapy, as well as the mining of related natural products and their potential roles, is of great importance to further alleviate the side effects of cancer therapies and improve the cure rate. In this paper, we will systematically review the relationship between gut microbial metabolites and tumor immunotherapy, and mine related natural products to explore their potential challenges and directions.

## 2 Sources and classification of gut microbial metabolites

Depending on the food source, gut microbial metabolites can be classified as SCFAs, bile acids, phenolics, vitamins, polyamines, tryptophan and lipids (21), which play an important role in tumorigenesis and progression (22–24). SCFAs are saturated fatty acids with chain lengths of one to six carbon atoms, which are the main product of fiber fermentation in the colon and are not absorbed in the small intestine (25), and are metabolized mainly by the phylum *Thick Walled Bacteria*, including *Fusobacterium harryi*, *Fusobacterium rectum* and *E. fecalis prausnitzii*, *Clostridium* sp*orotrichum*, *Bifidobacterium bifidum*, *Propionibacterium* sp, *Pseudomonas* sp and *Lactobacillus* sp (26, 27). SCFAs include mainly acetate, propionate and butyrate, with formate, valerate and hexanoate being the least abundant (28, 29), of which butyrate is the most studied SCFA, which not only maintains the balance of the intestinal flora, but also has an inhibitory effect on tumorigenesis, which may reduce the risk of colorectal cancer (30–33). Tryptophan is one of the essential amino acids and the only amino acid with an indole structure, which is found mainly in protein-rich foods such as poultry, fish, dairy products and soya beans. Studies have shown that tryptophan can be metabolized by the intestinal microflora to produce a variety of metabolites such as lactic acid, propionic acid, acrylic acid, tryptamine, etc., which may be involved in the immune response process (34). In addition, indole and indole metabolites produced by bacterial pathways, including indole-3-acetic acid (IAA), indole-3-aldehyde (IAld), indole-3-propionic acid (IPA), indole-3-acetamide (34–36), which can act as activators of aryl hydrocarbon receptor (AHR) or pregnane X receptor (PXR) signaling and play an important role in the regulation of the host immune response (37). AHR or PXR signaling activators play key roles in regulating immune responses, metabolic processes, and cellular responses to environmental signals (37, 38). Most of the intestinal bacteria involved in metabolism are of the Bacillus genus. *Escherichia coli* (*E. coli*) metabolizes tryptophan to indole and pyruvate (39), and *Bacillus* sp*haericus* metabolizes tryptophan to indole, which is further metabolized to form IAld, which can be oxidized to IAA by oxidoreductases or converted to tryptamine by decarboxylases. Inosine is mainly derived from endogenous purine nucleosides formed from the deamination metabolism of the adenine portion of nucleic acid by intestinal microorganisms and has the function of regulating the intestinal microbiota and protecting the intestinal mucosal barrier (40). Trimethylamine oxides are derived from the large amounts of choline or carnitine present in foods such as fish, eggs and meat products, which can be metabolized by the intestinal microflora *Fusobacterium*, *Anaplasma phagocytophilum*, *Aeromonas phagocytophilum*, *Clostridium phagocytophilum* and *Aspergillus phagocytophilum* to produce trimethylamine (TMA), which passes through the portal circulation to the liver where it is catalyzed to produce trimethylamine oxide (TMAO) (41). Secondary bile acids are converted from primary bile acids through metabolism by the gut microbiota, whereas primary bile acids are synthesized in the liver and are involved in fat metabolism in the hepatointestinal circulation (42, 43). Polyphenols are water-soluble organic compounds found in fruits, vegetables and herbs, and metabolites of polyphenols in the gut flora, such as phenolic acids and flavonoid derivatives, have been shown to directly or indirectly influence cancer development by stimulating the host immune response and reducing oxidative stress and inflammation (44).

## 3 The basic principles of tumor immunotherapy

The immune system plays a continuous role in surveillance and protection in the human body. During the early stages of tumor development, a large number of white blood cells, including various subsets of T cells and dendritic cells, typically accumulate in the TME. These cells can specifically recognize and eliminate tumor cells (45). However, during tumor development, multiple mechanisms of immune evasion and suppression evolve, including altering tumor antigens to prevent recognition by T cells (46, 47), blocking T cell recruitment to the TME (48), and utilizing immunosuppressive white blood cells such as Tregs and macrophages (49). *James Allison* and *Tasuku Honjo*'s discovery of T-cell inhibitory signaling pathways has brought immunotherapy to widespread attention. When these pathways are activated, they prevent effective cancer immunization.

### 3.1 Immune checkpoint inhibitors

Immune checkpoint inhibitors (ICIs) can effectively restore or enhance the reactivity of anti-tumor T cells, preventing immune escape by cancer cells. The most prominent of these are cytotoxic T-lymphocyte-associated protein 4 (CTLA-4) monoclonal antibodies and programmed cell death protein 1 (PD-1) monoclonal antibodies. CTLA-4 is a protein receptor expressed on T cells, which binds to CD80 and CD86 on dendritic cells with higher affinity than CD28 (50). This binding triggers intracellular inhibitory signaling that leads T cells into a state of non-responsiveness (51). CTLA-4 monoclonal antibodies can block the immunosuppressive effects of CTLA-4, reducing Treg cells and increasing effector T cells (52). In several animal model experiments, blocking CTLA-4 has been shown to effectively enhance anti-tumor effects (53, 54). PD-1 is a surface receptor for its homologous ligands programmed cell death ligand 1 (PD-L1) and programmed cell death ligand 2 (PD-L2). It is primarily expressed on activated T cells, but also present on other white blood cell subsets, including activated B cells, dendritic cells (DC), monocytes, and natural killer (NK) cell (55, 56). PD-L1 is found on activated T cells, B cells, dendritic cells, macrophages, and many tissue cells (57), whereas PD-L2 is only expressed on dendritic cells, macrophages, and some specialized cells (57). PD-1 can inhibit T cell activation through multiple pathways. In effector T cells, PD-1 activation leads to the dephosphorylation of key signaling molecules downstream of the T cell receptor (TCR), thereby suppressing TCR-mediated T cell activation (58). Other studies have shown that the PD-1/PD-L1 interaction preferentially triggers dephosphorylation of CD28, which is one of the main mechanisms of T cell suppression (59). PD-1 also enhances T cell motility, hindering T cell interactions with dendritic cells. Additionally, it has been reported that PD-1 can bind to CD80 and potentially compete with CD28 like CTLA-4, thereby inhibiting T cell activation. Cancer cells can utilize the PD-1 pathway to upregulate its ligands, PD-L1 and PD-L2, to inhibit T cell-mediated apoptosis. Multiple studies have been shown that upregulated PD-1 expression on tumor-infiltrating lymphocytes is associated with poor prognosis in many human cancers (60). CTLA-4 and PD-1 provide key physiological immune regulatory mechanisms, and extensive experimental data have shown that blocking these checkpoints with antibodies can enhance anti-tumor immune responses in animal models. This discovery has led to the development of humanized monoclonal antibodies targeting these molecules. These novel immunotherapies are known as ICIs. In 2011, the U.S. approved the first CTLA-4 inhibitor, Ipilimumab, for the treatment of metastatic melanoma (61). In a phase III clinical trial, 600 melanoma patients previously treated with other therapies were divided into three experimental groups: Ipilimumab combined with the gp100 vaccine, Ipilimumab alone, and gp100 alone. The results showed that the combination therapy group had the longest median overall survival (OS) (62). Currently, ICIs are widely used in cancer immunotherapy and have been approved for melanoma, non-small cell lung cancer (NSCLC), renal cell carcinoma, bladder cancer, and head and neck squamous cell carcinoma. Although ICIs can activate T cells and induce durable anti-tumor responses, this treatment can also lead to specific immune activation in non-tumor organs, causing immune-related adverse events (irAEs). Some adverse events, such as rashes, rheumatism, and others, are not life-threatening but can reduce the patient's quality of life, while other irAEs, such as pneumonia, myocarditis, hepatitis, and irAEs affecting the nervous and hematologic systems, can be fatal (63). *Kyoko* et al. reported a case of a metastatic breast cancer patient receiving combination treatment with Atezolizumab and paclitaxel, who developed a rare (1%) neurological irAE, leading to severe, permanent peripheral neuropathy (64). Furthermore, ICIs largely rely on the pre-existing presence of tumor-infiltrating cytotoxic T lymphocytes to exert their anti-tumor effects. In some patients, the immune cell concentration in the TME is low, and even when activated, the immune response is minimal.

### 3.2 Chimeric antigen receptor–T cell therapy

CAR T cell therapy has opened a new era in cancer treatment (65). Synthetic CAR constructs typically include an extracellular domain derived from the single-chain variable fragment of an antibody, as well as a hinge and transmembrane domain (53). The functional end of CAR usually contains activation and co-stimulatory domains, known as intracellular domains (66, 67). Commonly used co-stimulatory molecules include CD28 and 4-1BB. When the antigen activates this receptor, it triggers TCR signaling, co-stimulatory signaling, and cytokine signaling. The synergistic transmission of these three signals fully satisfies T cell activation and proliferation (68). CAR-T cell therapy has achieved tremendous success in treating B cell malignancies, including leukemia and lymphoma. The CAR-T cell therapies Tisagenlecleucel and Axicabtagene ciloleucel were approved by the FDA and the European Medicines Agency in 2017 and 2018, respectively, for the treatment of acute lymphoblastic leukemia, targeting the CD19 antigen on B cells (69). In 2021, the FDA approved the first cellular therapy for multiple myeloma (MM), idecabtagene vicleucel (70). CAR-T cells have also shown remarkable effects and prospects in lymphoma treatment. Axi-Cel, a CD19+ CAR-T cell therapy, has demonstrated significant efficacy in refractory large B cell lymphoma, showing good response levels in 28 treated patients (71). This drug was approved for the treatment of follicular lymphoma (FL), as 94% of the 80 evaluable FL patients in clinical trials showed a response, with 79% achieving complete remission (CR) (72). The FDA approved the CAR-T cell therapy Breyanzi® for the treatment of mantle cell lymphoma (MCL), which has been shown to be effective and safe in phase II clinical trials in patients with relapsed/refractory MCL (73). Despite the significant progress has been shown in CAR-T cell therapy clinical trials, the presence of adverse events during treatment has affected its effectiveness. The most common is off-target effects, where the same target antigen is expressed on normal cells, causing CAR-T cells to attack healthy tissue, leading to adverse reactions. Notably, most of these toxic reactions are reversible if the patient receives timely intervention or treatment (74). Antigen escape is another major challenge faced by CAR-T therapy. Tumor cells escape killing by promoting mutations in genes encoding antigens, leading to downregulation or loss of the alternative antigen that lacks CAR-T cell targeting epitopes (75). For example, although 70% to 90% of relapsed or refractory ALL patients exhibit long-term responses to CD19-targeting CAR-T cell therapy, recent follow-up data indicate a common resistance mechanism, with 30% to 70% of patients who relapse after treatment showing downregulation or loss of the CD19 antigen (76). Furthermore, in the solid TME, immune suppression and fibrosis greatly limit the targeted therapeutic effects of CAR-T cells. To date, no CAR-T cell therapy has been approved for solid tumors.

### 3.3 Therapeutic vaccines

Therapeutic cancer vaccination is an active immunization strategy aimed at stimulating adaptive immune responses against tumor antigens and generating tumor-specific functional immune effector cells, such as cytotoxic T lymphocytes (77). Broadly, cancer vaccines can be classified into cell-based vaccines, viral vector vaccines, and molecular vaccines composed of peptides, deoxyribonucleic acid (DNA), or ribonucleic acid (RNA). Cancer vaccines targeting cells use non-replicating cancer cells or antigen-presenting cells (APCs) carrying cancer antigens (78). Sipuleucel-T (Provenge®) is the first FDA-approved therapeutic cancer vaccine, which improved OS in patients with metastatic castration-resistant prostate cancer in the Phase 3 trial (79). Cancer vaccines can mimic natural immune processes, demonstrating good safety and therapeutic efficacy in numerous clinical trials (80). In an early study, *Hoover* et al. showed survival benefits of autologous tumor cell vaccines in colon cancer, highlighting the broad potential of cancer vaccines (80). In melanoma, *Mackiewicz* et al. observed the highest proportion of complete responders in a trial using allogeneic whole-cell vaccines (81). In 2020, *Lv* et al. conducted a Phase II randomized controlled trial of DC vaccines glioblastoma multiforme (GBM) and observed improved survival rates (82). *Yamanaka* et al., *Cho* et al., and *Yao* et al. compared the effects of dendritic cell vaccines and conventional therapies in glioblastoma patients, with the vaccine treatment group showing significant survival benefits (83). However, it must be acknowledged that the anticipated efficacy of cancer vaccines has not been perfectly realized in clinical settings. Despite extensive preclinical and clinical work, their effectiveness remains unpredictable, and there are significant negative feedbacks.

## 4 Crosstalk between gut microbial metabolites and tumor immunity

**Figure 1:**
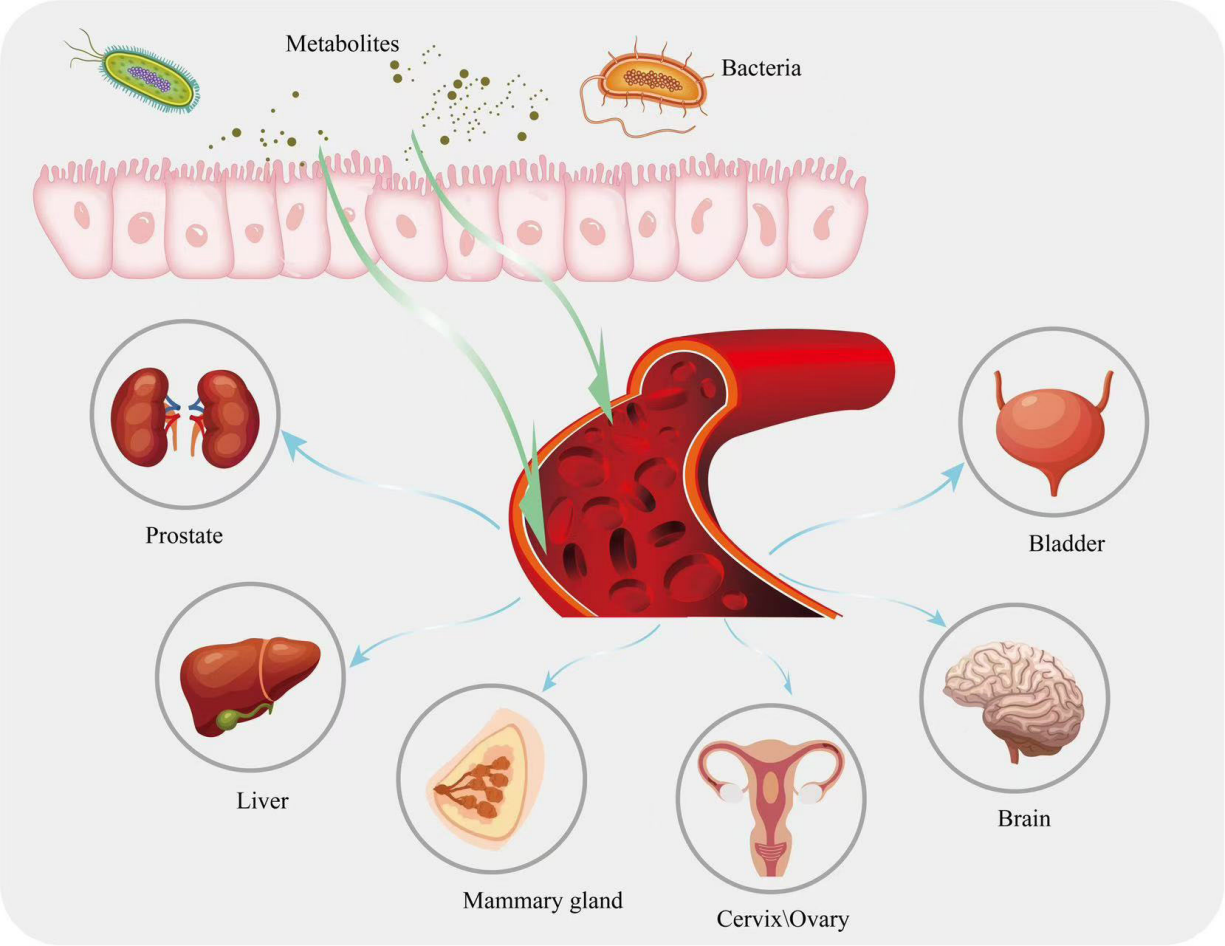
Metabolites in the intestine are circulated to other parts of the body along with the bloodstream.

Gut microbial metabolites can diffuse across the epithelium and lamina propria into the somatic circulation, and studies have shown that a large number of microbial molecules have been detected in the human bloodstream, of which 5-10% are derived from the gut microbiota, and that these microbial molecules may act as regulators of cellular functions in distal organs (84, 85). In addition, gut microbial metabolites can enter the circulation along the gut-X axis to reach other target organs or target cells, act as human hormone-like signaling mediators to influence the homeostasis of the local environment (86–90), participate in the release of cytokines in the TME and in the development and differentiation of immune cells, thereby inhibiting immune escape from tumors and indirectly influencing the response to various classical immunotherapies (91–95) (**Figure 1**).

### 4.1 Immunoregulation of the gut-liver axis

Studies have shown that the gut microbiome of patients with liver pathology differs significantly from that of healthy individuals (96), and gut microbiology and liver function have been found to be linked to physiological anatomy. Fecal microbiota transplantation (FMT) using fecal samples from patients with hepatitis C virus-associated chronic liver disease was found to promote liver tumor growth in mice (97). *Ponziani* et al. investigated the changes in microbial populations in patients with hepatocellular carcinoma (HCC) as well as in healthy patients and found that the abundance of *Eosinophilus* spp., *Enterococcus* spp., *Streptococcus* spp., *Dictyococcus* spp. and *Cholera* spp. were higher in HCC patients than in controls, and that there was a decrease in *Bifidobacterium Akerman*, *Bifidobacterium bifidum* and *Bifidobacterium minutissimus* spp. and an increase in plasma interleukin-8 and interleukin-13 (98). Interestingly, gut microbes and their metabolites play a crucial role in maintaining the physiological state and metabolic homeostasis of the host. For example, *Clostridium* difficile metabolically alters the balance of primary and secondary bile acids, which in turn induces the production of C-X-C motif chemokine ligand 16 (CXCL16) in liver sinusoidal endothelial cells, and CXCL16 can induce NK cells to accumulate in the liver and generate anti-tumor immunity (99). In addition, deoxycholic acid (DCA) induces hepatic stellate cells to express a senescence-associated secretory phenotype, which promotes immune expression to eliminate senescent cells and induces fibrosis and carcinogenesis (100, 101). In addition, inulin can produce anti-inflammatory SCFAs through intestinal microbial metabolism, and oral inulin administration promotes CD8^+^ T-cell expression and enhances the therapeutic effect of PD-1 checkpoint immunosuppressants (102). *Lee* et al. (103) conducted a fecal analysis of 41 patients with unresectable liver cancer, including 20 ICI responders and 21 non-responders. The results revealed that the concentration of ursodeoxycholic acid (UDCA) in the feces of ICI responders was significantly higher, while the concentration of lithocholic acid was increased in the feces of patients. This suggests that bile acids are associated with the response of HCC patients to ICI treatment. *Han* et al. (104) found that the intestinal microbial metabolite D-lactic acid (DL) reaches the liver via the portal vein and converts tumor-associated macrophages (TAMs) from M2 to M1, enhancing the phagocytic function of Kupffer cells. The mechanism involves the inhibition of the phosphoinositide 3-kinase (PI3K)/protein kinase B (Akt) pathway and the activation of the nuclear factor kappa B (NF-κB) pathway. *Zheng* et al. (105) performed dynamic analysis of HCC patients receiving anti-PD-1 immunotherapy in the 6th week and found significant differences in the gut microbiome diversity between immune responders and non-responders. The fecal samples of immune responders showed higher taxonomic richness and gene counts, enriched in Myxobacteria and Ruminococcaceae, which influenced the efficacy of anti-PD-1 immunotherapy and disease prognosis in HCC patients. Dysbiosis of the gut microbiome can also impact resistance to immunotherapy. *Arielle* et al. (106) demonstrated that antibiotic-induced dysbiosis reduces ICI activation of the immune system, leading to resistance and poor treatment response. Vetizou (107) showed that *Bacteroides fragilis* and *Bacteroides thetaiotaomicron* can regulate specific T cell responses. FMT from ICI responders helped non-responders recover the anti-cancer effects of PD-1 and CTLA-4 immune checkpoint blockade (108).

### 4.2 Immunomodulation of the gut-endocrine axis

The gut microbiota is recognized as an endocrine organ capable of influencing distal organs and related biological pathways. Dysbiosis of the gut microbiome can lead to diseases such as breast, cervical and ovarian cancer in women, and gut microbial metabolites are important mediators of associated diseases (109). Gastrointestinal flora can influence non-ovarian estrogen levels via the hepatointestinal cycle (110), for example, butyrate has been shown to regulate luteinizing hormone and estradiol secretion via the cyclic adenosine monophosphate (cAMP) signaling pathway (111). *Liu* et al. showed that butyrate supplementation alleviated non-alcoholic fatty liver disease in ovariectomized mice (112). Another study showed higher levels of *Prevotella* in healthy women compared to breast cancer patients (113). *James* et al. (114) also demonstrated that the diversity of the gut microbiome is reduced in breast cancer patients, with an increased relative abundance of *Clostridia*. *Miko* and *Luu* et al. (115, 116) found that the reduction in microbial diversity is most pronounced in early-stage breast cancer (Stage 0 and Stage 1). The reduction in gut microbiome diversity directly affects the levels of metabolic products and increases the incidence of breast cancer (117). In a mouse study, the use of ampicillin exacerbated the reduction in gut microbiome diversity and induced tumor formation, which may be related to the decreased abundance of *Odoribacter* and *Anaeotruncus*, both of which are bacteria that produce butyrate (118). In addition, *Mikoah* et al. found that lithocholic acid (LCA) inhibited the proliferation of MCF7, SKBR3, and 4T1 breast cancer cells, and tested the cytostatic properties of LCA in mice transplanted with 4T1 breast cancer cells, and found a significant reduction in the ability of the primary tumors to infiltrate the surrounding tissues and metastasize after LCA treatment, which suggests that LCA can be transferred through the bloodstream to the breast and may play an important role in promoting the antiproliferative effects of breast cancer (119). In addition to affecting tumor progression, gut microbiome metabolites also influence the responsiveness to immunotherapy. In a human study, strains were extracted from the feces of breast cancer patients who responded and did not respond to trastuzumab treatment. Mice receiving FMT from responders also showed a response to trastuzumab, whereas those receiving FMT from non-responders did not. This was associated with a higher abundance of *Clostridia* in the gut of trastuzumab responders, which is capable of producing SCFAs (120).

### 4.3 Immunomodulation of the gut-brain axis

The complex relationship between the gut microbiota and host health can be further understood through the gut-brain axis (GBA) and its associated biological activities. Gut microbial metabolites can cross the blood-brain barrier through the body's circulation and, because of their small molecular size, play an immunomodulatory role in the gut-brain axis. Gut microbial tryptophan metabolites can signal to the brain, suggesting a potential role for metabolites in communication between gut microbes and the central nervous system (CNS) (121, 122), and indole derivatives act as AHR ligands, including IPA and IAA, and have the ability to cross the blood-brain barrier, giving them a key role in the GBA. For example, indole, IPA and IAld can activate AHR signaling in astrocytes and suppress inflammation in the CNS (123). IPA has a strong free radical scavenging and antioxidant capacity, protecting primary neurons and neuroblastoma cells from oxidative damage (124). Circulating SCFAs produced by gut microbiota metabolism affect the integrity of the blood-brain barrier by increasing the production of tight junction proteins, and increased blood-brain barrier integrity reduces the entry of unwanted metabolites into brain tissue and enhances blood-brain barrier defense mechanisms (125). Compounds produced by gut microbiota metabolism, such as lipoproteins and lipopolysaccharides, affect autoimmune function by stimulating immune cells to release cytokines that can cross the blood-brain barrier and activate neurons, altering neurological function and leading to mood and behavioral changes (126). *Zhou* et al. used an *in situ* GBM model and found that gut dysbiosis leads to an increased proportion of M2-like macrophages in the TME. They also observed reduced levels of SCFAs in the blood and glioma tissues of the brain. Oral supplementation with SCFAs induced the differentiation of macrophages into the M1 type in the TME, enhancing the immune response. The mechanism may be related to SCFA activation of glycolysis in macrophages (127).In a study investigating the changes in the gut microbiota of brain tumor patients and the association between the two, 16S rRNA gene amplicon sequence sequencing was used to characterize the gut microflora in 158 patients, and the results showed that the abundance and homogeneity of the gut microbial ecosystem in brain tumor patients was significantly lower than that in healthy controls, as evidenced by an increase in the abundance of pathogenic bacteria and a decrease in the abundance of probiotic bacteria.

### 4.4 Others

Several studies have shown that gut microbial metabolites also play a critical role in immune modulation in many other types of tumor disease. For example, in one study, supplementation of mice with long-chain fatty acid metabolizing enzymes to reduce levels of butyrate metabolites reduced prostate cancer (PCa) growth and worsening of PCa in mice prior to prostatectomy, demonstrating a cross-talk between the gut microbial metabolite butyrate and prostate cancer (128). Butyrate, as one of the main components of SCFAs, can reduce the risk of pancreatic ductal adenocarcinoma (PDAC), inhibit the proliferation of pancreatic cancer cells, and induce their differentiation into a secretory phenotype with ultrastructural changes. Studies have shown that hyaluronic acid conjugates of butyrate can significantly inhibit cell proliferation in pancreatic cancer cell cultures (129).Another study showed that inosine improved the efficacy of monoclonal antibodies against CTLA-4 and against PD-L1 in mouse models of bladder and small bowel cancer (130). *Liu* et al. (131) conducted gut microbiome analysis, including metagenomic and metabolomic sequencing, on 54 lung cancer patients who initially received PD-1/PD-L1 treatment. They found that patients with higher levels of acetate, propionate, and butyrate had longer progression-free survival and lower tumor progression risk, suggesting a crosstalk between gut microbial metabolites and the lungs. *Andrea* et al. (132) characterized the metabolic features of the gut microbiome in 11 NSCLC patients treated with the PD-1 monoclonal antibody, nivolumab. They found that 4 patients with early progression were significantly associated with the gut metabolites 2-pentanone and tridecane, whereas propionate and butyrate in SCFAs were linked to long-term beneficial outcomes in the remaining 7 patients. *Motoo Nomura* et al. (133) measured SCFA concentrations in feces and plasma from 52 patients with solid tumors receiving PD-1 treatment and found that high concentrations of acetate, propionate, butyrate, and valerate in feces were significantly associated with longer progression-free survival. SCFAs exhibited immune-modulatory functions in the host, possibly through the inhibition of histone deacetylases (HDAC) (134). Two phase I clinical trials showed that FMT could improve response to ICIs in resistant metastatic melanoma (91). Additionally, choline metabolites TMAO and TMA were found to enhance PDAC response to immune checkpoint blockade and improve survival in tumor-bearing mice. *Gauri Mirji* et al. treated PDAC model mice with macrophages induced by TMAO and found that tumor burden was reduced by more than 2.4 times compared to mice receiving control macrophage treatment, with significant upregulation of Interferon-*γ*, CD103, and CD44 on CD8+ and CD4+ cells. This suggests that TMAO induces macrophages to differentiate into a phenotype that enhances T-cell effects and suppresses PDAC growth. Similar effects were observed in human macrophages. The authors then used TMAO in combination with PD-1 or Tim-3 antibodies to treat PDAC model mice and found that, compared to the PD-1 and TMAO monotherapy groups, the combination therapy enhanced the immune activation status in the PDAC TME, with stronger activation of bone marrow cells and T cells (135). Gut microbial metabolites not only improve cancer immunotherapy prognosis by enhancing immune responses, but also alleviate immune-related adverse effects. For example, CTLA-4 antibody treatment is often associated with gut-related side effects, such as diarrhea or colitis, while PD-1/PD-L1 antibodies are more commonly associated with thyroid dysfunction or pulmonary toxicity. As a result, many patients can only use these treatments for a short period of time. SCFAs, as nutrients for intestinal epithelial cells, can help repair the gut mucosa, reduce gastrointestinal adverse effects, and help extend the treatment cycle of CTLA-4. Oral probiotics such as *Bacteroides fragilis* and *Burkholderia cepacia* can also help alleviate adverse effects (108). The impact of gut microbial metabolites on tumor immunity is not always beneficial and can sometimes promote the occurrence and progression of cancer. For example, secondary bile acids such as DCA and LCA, which are metabolized by gut microbes, are associated with the proliferation, survival, and metastasis of cancer cells (129). In rodent model experiments, it has been shown that elevated serum concentrations of DCA and LCA increase the risk of liver cancer (136). The mechanism is related to the activation of nuclear receptors such as FXR and PXR, which lead to changes in gene expression (137). *Clélia Coutzac* et al. (138) used a CT26 tumor model in mice treated with intraperitoneal CTLA-4 antibody and administered sodium butyrate in their drinking water to observe the effects of SCFAs on the anti-tumor effect of CTLA-4. The results showed that there was no significant reduction in tumor growth compared to the control group, suggesting that butyrate may inhibit the anti-tumor efficacy of CTLA-4 in mice. The underlying reason may be related to its impact on dendritic cell maturation and T-cell function, although the exact mechanism still needs further exploration. Gut microbes and their metabolites have a double-edged sword effect on tumor immunotherapy. Under the influence of factors such as diet, medication, and disease, the balanced distribution of gut microbiota is disrupted, leading to an imbalance in the types of metabolites. This change can affect gut mucosal function, increase intestinal permeability, and allow a large number of small molecular metabolites to pass through the intestinal wall, enter the bloodstream, and accumulate in the TME. These metabolites then regulate immune function, potentially having either positive or negative effects on tumor immunotherapy. Numerous metabolites have been confirmed to play a role in tumor development. By orally supplementing or transplanting isolated strains, it is possible to artificially intervene in metabolite levels and influence the effectiveness of tumor immunotherapy. This also opens up new avenues for cancer drug development.

## 5 Mechanisms by which gut microbial metabolites influence tumor immunity

### 5.1 Regulation of the innate immune response

The gut microbial tryptophan metabolite indole and its derivatives can directly regulate the growth and differentiation of non-specific immune cells through activation of the AHR pathway (139). For example, it has been shown that IAld and IAA promote the differentiation of monocytes into dendritic cells and enhance the phagocytic activity of neutrophils and macrophages (140, 141), and that tryptophan activates the AHR pathway in NK cells, which enhances cytotoxicity against tumor cells (8, 142, 143). Gut microbial tryptophan metabolites can also mediate the inflammatory response by regulating cytokine expression, in particular stimulating a key role for macrophages. A study in mouse liver showed that IAA attenuated high fat diet-induced hepatotoxicity and inhibited interleukin-1β (IL-1β), interleukin-23 (IL-23) and tumor necrosis factor-α (TNF-α), monocyte chemoattractant protein-1 (MCP-1) in a dose-dependent manner, interleukin-17A (IL-17A), interleukin-6 (IL-6), and other inflammatory factors, while increasing the levels of the anti-inflammatory factor interleukin-10 (IL-10) and decreasing the pro-inflammatory factor/anti-inflammatory factor ratio (37, 144, 145). Indole also has a similar function in attenuating the expression of key proteins in the NF-κB pathway, thereby inhibiting the expression of inflammatory factors while increasing the expression of anti-inflammatory factors (146, 147), inhibiting the inflammatory response and promoting tumor cell growth and metastasis (148). Tryptophan reduces TNF transcription by mediating a decrease in IL-6 signaling capacity via AHR targets (149), whereas IAA neutralizes free radicals, thereby attenuating the inflammatory response of RAW264.7 macrophages to lipopolysaccharide (LPS) and increasing interleukin-8 (IL-8) signaling (150), and similarly, indole-3-methanol inhibited LPS-mediated inflammatory cytokine production in mouse bone marrow-derived macrophages (BMM) (151). Studies have shown that propionic acid and butyric acid in SCFA can inhibit HDAC and increase histone acetylation to play an anti-inflammatory and anti-cancer role (126). In HCC, gut microbial metabolites, including lipoteichoic acid (LTA) and DCA, upregulate cyclooxygenase-2 (COX-2) expression via toll-like receptor 2 (TLR2) on the membrane surface of tumor cells, which increases prostaglandin E2 (PGE2) expression. Activation of the COX2-PGE2 pathway inhibits DCs, NK and T cells, which promotes immune escape and affects the efficacy and prognosis of tumor immunotherapy. TAMs are the major type of infiltrating immune cells in the TME (152). In a study by *Parida S* et al. in a mouse model of breast cancer, the number of pro-tumorigenic M2-like macrophages was significantly increased in advanced tumors of mice with gut microbiota dysbiosis compared to non-ecological dysbiosis controls with the same tumor burden (153), suggesting that gut microbes can induce macrophage polarization to either M1 or M2, thereby altering the TME and promoting or hindering the effects of tumor immunotherapy. LPS, a metabolite of *E. coli*, can induce M2 polarization of TAMs, thus leading to rapid cancer development, for example, in the colorectal cancer (CRC) mouse model, with the increase of *E. coli*, its metabolite LPS can upregulate the secretion of cathepsin K by CRC cells, which can induce M2 polarization of TAMs, leading to rapid CRC development (154), while a high-fat diet can induce gut microbiota dysbiosis, reduce SCFA levels, activate the MCP-1/C-C motif chemokine receptor 2 (CCR2) axis, and promote TAMs recruitment and polarization towards M2, ultimately leading to CRC development (155).

### 5.2 Regulation of adaptive immune responses

Numerous studies have shown that the gut microbial tryptophan metabolite indole and indole derivatives can regulate Treg/T helper cell 17 (Th17) differentiation and thereby suppress the inflammatory response (156). For example, indole-3-carbinol (I3C) treatment reduces colorectal cancer by decreasing Th17 cells and increasing Treg (109). In addition, I3C increased the production of CD4^+^Foxp3^+^ cells and ultimately reduced CD4^+^IL-17^+^ cells that induce neuroinflammation in mice, and IAA and IPA promote Treg differentiation and function by upregulating the expression of Foxp3 (Forkhead Box P3) and other Treg-related genes (37, 41, 157). IAA promotes Treg differentiation by activating the AHR pathway (37). In addition, tryptamine has been shown to activate the mechanistic target of rapamycin (mTOR) in Treg cells *in vitro*, to increase the expression of phospho-eukaryotic translation initiation factor 4E binding protein 1 (P4EBP1), and to increase the expression of phosphorylated ribosomal protein S6 kinase 1 (P-S6K1) in Tem cells (158), suggesting that exogenous tryptophan promotes cytotoxic T cell glycolysis, thereby affecting the regulation of CD4^+^ T cell function (159). In addition to the effects of IPA on T lymphocytes, indole derivatives have been shown to inhibit and stimulate B cells. I3C can regulate B cell function by inhibiting the production of the immunoglobulins immunoglobulin M (IgM) and immunoglobulin G (IgG) and decreasing the expression of the cell surface antigen CD69. CD69, inducing B cell apoptosis and inhibiting B cell proliferation (160, 161). Tryptamine has been shown to stimulate IgA production by B cells and activate the transcription factor AHR to regulate B cell function (162). Although several studies have demonstrated the inhibitory and stimulatory effects of indole derivatives on B cells, further research into the mechanism of action of these compounds is needed before they can be used in the potential treatment of immune diseases.

Gut microbial bile metabolites also play an important role in T cell expression. DAC has been shown to inhibit the Ca^2+^-nuclear factor of activated T cells-2 (NFAT2) signaling pathway by targeting the plasma membrane Ca^2+^ATPase (PMCA), and in CRC patients, the effective function of CD8^+^ T cells is negatively correlated with DCA concentration and expression (163). Another study showed that the tryptophan metabolite indole activates the AHR and also promotes the conversion of TAMs to an immunosuppressive phenotype in pancreatic ductal adenocarcinoma and inhibits the accumulation of CD8^+^ T cells in the tumor (9). Researchers have found that selective depletion of TAMs in a mouse model of breast cancer increases CD8^+^ T cell infiltration into the TME and stimulates anti-tumor immune responses (164, 165).

Specific types of gut metabolites, including SCFA, tryptophan, etc., show a compelling role in cancer inhibition. This is because these compounds show significant activity on immune signaling and cell division processes (95). SCFA recognizes specific G protein-coupled receptors (GPCRs) on the surface of immune cells, including GPR41 and GPR43, which leads to an increase in the total number of regulatory T cells, transforming growth factor-β1 (TGFβ1) and levels of the anti-inflammatory cytokine IL-10 in the host (166). An inhibitory effect on T-cell mediated autoimmune responses was also found with diets high in SCFA. In contrast, another study found that *Lactobacillus* metabolizes dietary tryptophan to indole, which activates the AHR and inhibits the accumulation of CD8^+^ T cells in tumors. In human CRC, the gut microbiota stimulates tumor cells to produce chemokines to recruit anti-tumor T cells into tumor tissue and play an immune role (167).

**Figure 2:**
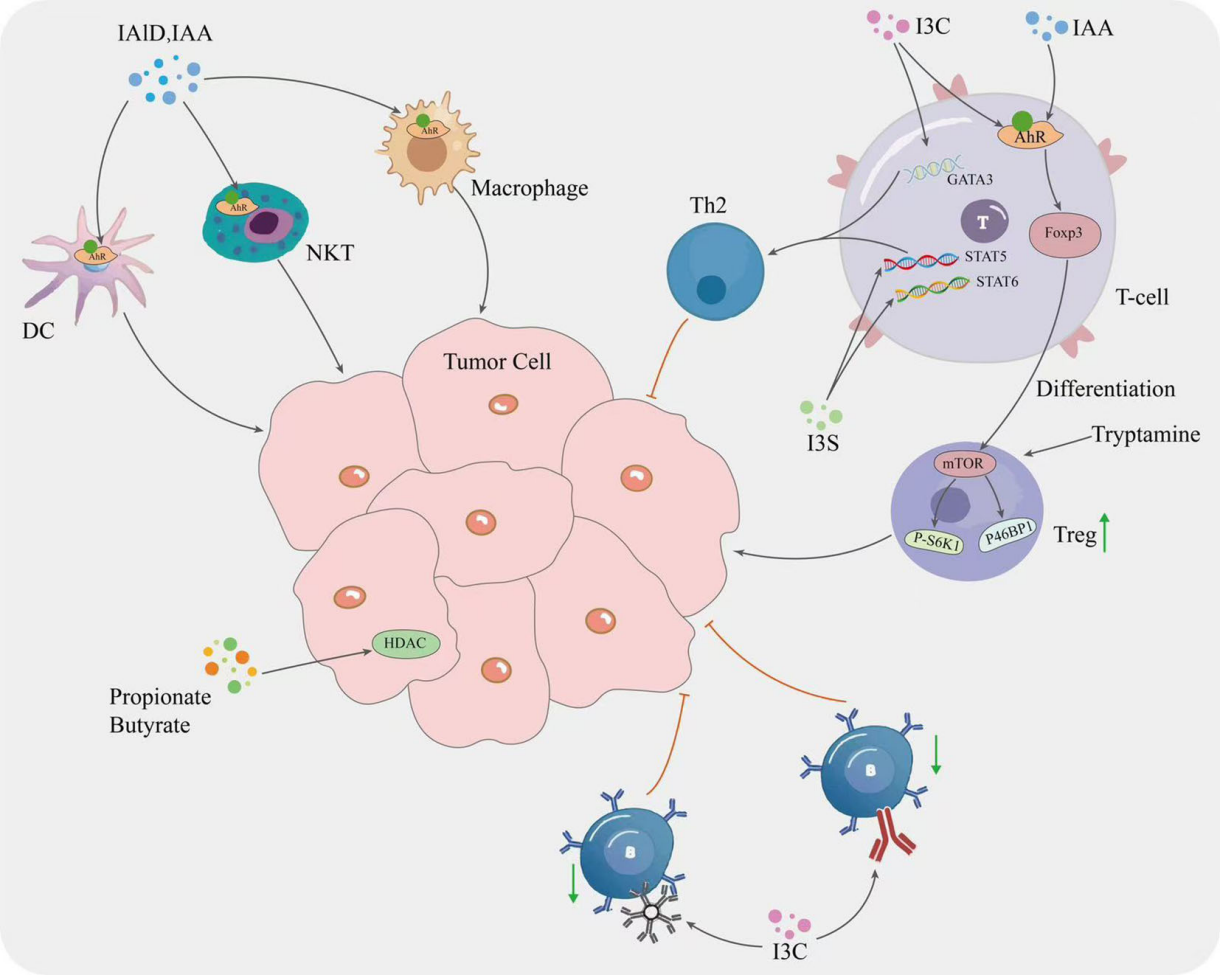
Mechanisms by which gut microbial metabolites influence tumor cell.

Indole derivatives can regulate T cell differentiation by modulating the expression of cytokines and transcription factors. For example, the indole derivative I3C has been shown to promote Th1 cell differentiation by upregulating interferon gamma and Tβ transcription factors (161). In addition, I3C inhibits T helper 2 cell (Th2) differentiation by downregulating the expression of IL-4 related genes and the transcription factor GATA binding protein 3 (GATA3) (161). Another indole derivative, Indoxyl sulfate (IS), has also shown the ability to regulate T cell activation and proliferation. IS inhibits the expression of CD25 and CD69 surface markers (162), which are key indicators of early T cell activation (168, 169). In addition, IS reduced the frequency of IL-4-producing CD4^+^ T cells and inhibited Th2 differentiation. This effect was attributed to inhibition of phosphorylation of signal transducer and activator of transcription 5 (STAT5) and signal transducer and activator of transcription 6 (STAT6), transcription factors involved in Th2 differentiation. Indoxyl 3-sulfate (I3S) can also induce T cell apoptosis or programmed cell death by upregulating the expression of pro-apoptotic proteins (170). Other indole derivatives IAld and IPA have also been shown to modulate the immune response by regulating T cell function (171, 172). IAld induces T-cell apoptosis and inhibits T-cell activation by regulating the expression of pro- and anti-apoptotic proteins (173) (**Figure 2**).

## 6 Potential application of natural products to microbial metabolites in tumor immunotherapy

Natural products have unique advantages such as a wide range of sources, low side effects and diverse biological activities. Natural products not only have broad anti-tumor activity (174). They also have the ability to modulate complex host-microbe interactions. Furthermore, an increasing amount of evidence indicates that natural products can influence the immune microenvironment of tumors by regulating microbial metabolites. Natural products can also target the microbiome to improve the efficacy of chemotherapy, overcome drug resistance and provide new therapeutic ideas and strategies for tumor immunotherapy (**Table 1**).

**Table 1:** Mechanisms of tumor therapy by natural products and their active ingredients via gut microbial metabolites.

Natural product	Active ingredient	Microbial metabolites	Mechanism	References
*Coptis chinensis*	Berberine	SCFAs	Inhibits intestinal inflammatory response and modulates colon epithelial cell signaling pathways in CAC mice via tryptophan metabolism and Wnt signaling pathway. Regulates intestinal microbiota imbalance, improves intestinal mucosal barrier function. Increases NK cell infiltration, increases levels of SCFAs, and activates mitochondrial apoptosis pathway as well as Fas death receptor apoptosis pathway for breast cancer.	(178) (179)
*Sophora flavescens*	Matrine	SCFAs	Increased SCFA levels in the intestine via the TLR4/NF-κB/MAPK signaling pathway.	(180)
*Pulsatilla chinensis*	Pulsatilla chinensis saponin	SCFAs	Increased levels of colonic SCFAs, activation of the GPR43-NLRP3 signaling pathway, and decreased levels of IL-1β, IL-6, and TNF-α pro-inflammatory cytokines.	(182)
*Panax ginseng*	Ginseng glucosyl oleanolate	SCFAs	Regulation of the MAPK signaling pathway inhibits Hep G2 cell proliferation, rebalances the intestinal flora, increases the level of total SCFAs improves intestinal inflammation and inhibits tumor growth.	(183)
*Gynostemma pentaphyllum*	Jiaogulan saponins	SCFAs	Improvement of the inflamed intestinal barrier, polarization of colonic M1 macrophages to M2 macrophages, positive restoration of the E-calmodulin/N-calmodulin ratio and down-regulation of oncogenic signaling molecules, and promotion of SCFAs-producing bacteria in a time-dependent manner.	(186)
*Brown algae*	Fucoidan	SCFAs	Regulate tryptophan metabolism and SCFAs to enhance CD8^+^ T cell function, increase the production of IFN-γ and TNFα, reduce the inhibitory effect of Treg in the circulatory system, remodel the intestinal microbiota, and cooperate with PD-1 monoclonal antibody to exert anti-tumor effects.	(187)
*Cichorium glandulosum*	Inulin	SCFAs	Regulation of intestinal flora, production of SCFAs, and inhibition of EMT transition processes.	(189)
*Tetrastigma hemsleyanum*	*Tetrastigma hemsleyanum* polysaccharide	SCFAs	Increased ileal secretion of immunoglobulin A and SCFAs restored tumor-induced intestinal microflora disturbances and promoted macrophage phagocytic index, NK cell activation.	(191)
*Tremella fuciformis*	*Tremella fuciformis* polysaccharides	Tryptophan metabolites (xanthurenic acid and kynurenic acid), Bile acids (DCA)	Stimulation of Foxp 3 ^+^ T cells promoted the production of anti-inflammatory cytokines, significantly increased intestinal flora diversity, and modulated tyrosine biosynthesis, tryptophan metabolism, and bile acid metabolism pathways.	(192)
*Kaempferia galanga*	Kaempferol	Bile acids (chenodesoxycholic acid)	Reducing tumor load, restoring the damaged intestinal barrier, down-regulating the secondary bile acid synthesis pathway, and increasing the protein expression of FXR to inhibit the activation of the Wnt/β-catenin pathway lead to the inhibition of CRC.	(194)
*Dioscorea esculenta*	Sitosterols	SCFAs	Decreases PI 3K/Akt expression, promotes Bad activation, decreases Bcl-xl, and increases cyto-c release, leading to caspase-9 and caspase-3 activation, PARP cleavage, and apoptosis. Increased levels of SCFAs led to apoptosis of cancer cells *in vitro*.	(195)
*Houpoea officinalis*	Magnolol	Tryptophan metabolites (kynurenic acid, 5-hydroxyindoleacetic acid, IAA, indolelactic acid and indoxylsulfuric acid)	Anti-inflammatory, restores tryptophan metabolites and enhances AHR activation inhibited by colonic inflammation thereby inhibiting colonic inflammation.	(196)
*Vaccinium macrocarpon*	Proanthocyanidins	Bile acids (chenodesoxycholic acid)	Targeting the gut microbiome-esophageal metabolome axis, modulating the TLR/NF-κB/TP 53 signaling pathway to inhibit EAC, and specifically reversing reflux-induced bacterial, inflammatory, and immune-related proteins and genes.	(198)

### 6.1 Alkaloids

In recent years, alkaloid chemicals extracted from plants have been found to have good anti-tumor activity, high efficacy, good tolerability and few toxic side effects (175). Berberine (BBR) is an isoquinoline alkaloid extracted from the roots and bark of plants in the *Coptis chinensis* Franch. Dextran sulfate sodium (DSS) can break down the intestinal mucosal barrier, leading to intestinal inflammation and ulcers. Previous studies have shown that BBR can ameliorate the bile acid imbalance induced by DSS in both the liver and the intestine. It achieves this by restoring the perturbed gut microbiota and activating the FXR and TGR5 signaling pathways, thereby alleviating DSS-induced ulcerative colitis in mice (176). Prolonged ulcerative colitis causes high levels of pro-inflammatory cytokines to accumulate in the colonic mucosa, and this accumulation, which in turn leads to proliferative lesions, is considered to be a major risk factor for the development of colorectal cancer (177). BBR was further investigated by *Wang* et al. and found to be therapeutically useful for potential protection against oxidative azomethane (AOM)/DSS-induced colitis and tumors in mice. Transcriptome analysis revealed that BBR regulates colonic epithelial cell signaling pathways in colitis-associated colon cancer mice through tryptophan metabolism and Wnt signaling pathways, which may affect fecal metabolites and SCFA metabolism (178). In addition, BBR also synergized with exercise to slow the progression of breast cancer in 4T1 hormone mice, and this synergistic effect was able to enhance the body's immune function, significantly increase levels of SCFAs, and activate the mitochondrial apoptotic pathway as well as the Fas death receptor apoptotic pathway, resulting in an anti-cancer effect (179). Furthermore, it has also been shown that Matrine increases SCFA levels in the gut and prevents intestinal damage through the Toll-like receptor 4 (TLR4)/NF-κB/mitogen-activated protein kinase (MAPK) signaling pathway, demonstrating its great potential as a potential therapeutic agent for the treatment of cancer (180).

### 6.2 Glycosides

Glycosides, as a class of natural products widely found in plants, have a variety of biological activities, mainly through the interaction with intestinal flora and metabolites to exert a medicinal effect, especially in the antitumor and immunomodulatory aspects showing significant potential (181). Furthermore, Pulsatilla chinensis saponin (PRS) is a natural saponin analogue isolated from *Pulsatilla chinensis* (Bunge) Regel. PRS protects against DSS-induced inflammatory bowel disease by increasing the levels of SCFAs, activating the GPR 43-NOD-like receptor thermal protein domain associated protein 3 (NLRP 3) signaling pathway and decreasing the levels of pro-inflammatory factors IL-1β, IL-6 and TNF-α *in vivo (* 182). Among these, *Panax ginseng* C. A. Mey. has been widely used as a functional food and medicine. Ginseng glucosyl oleanolate (GGO) was enzymatically prepared from ginsenoside Ro. GGO inhibited the proliferation of Hep G2 cells through phosphorylation of the MAPK signaling pathway, rebalanced the intestinal flora, increased the concentration of total SCFA, and increased the levels of acetic and propionic acids in the colon, thereby delaying the progression of liver tumors (183). Jiaogulan saponins are extracted from the dried above-ground part of Cucurbitaceae gynostemma, with hypoglycemic and anti-tumor effects (184, 185). *Imran Khan* et al. found that the combination of jiaogulan saponin and *Ganoderma lucidum* polysaccharides to improve the inflamed intestinal barrier in Apc^Min/+^ mice polarized colonic M1 macrophages to M2 macrophages, positively restored the E-calmodulin/N-calmodulin ratio and downregulated oncogenic signaling molecules, and increased the production of SCFAs in bacteria in a time-dependent manner (186).

### 6.3 Polysaccharides

Polysaccharides are macromolecular compounds widely found in plants, fungi, marine organisms and other natural sources, usually consisting of monosaccharides or oligosaccharides linked by glycosidic bonds, with diverse structures and rich biological activities. Polysaccharides can influence the tumor immune microenvironment by regulating the metabolic activity of the gut microbiota and increasing the synthesis of microbial metabolites.

Fucoidan, mainly derived from marine brown algae and marine invertebrates, is a sulfate -rich functional polysaccharide unique to the oceans that may enhance anti-PD-1 immunotherapy by modulating breast cancer-induced alterations in tryptophan metabolism and glycerophospholipid metabolism pathways, which are significantly increased by SCFAs, particularly acetic acid and butyric acid (187). They act as metabolic immunomodulators. They enhance the function of the T-lymphocyte immune response and anti-tumor immunity against breast cancer (188). Inulin increases the relative abundance of *Bifidobacterium*, *Lactobacillus* and *Leptospira* and restores the levels of acetic, propionic, isobutyric and butyric acids, thereby inhibiting the process of epithelial-mesenchymal transition (EMT) and inhibiting metastasis of colorectal cancer (189). Inulin is a natural soluble functional fructan. It has been found that inulin produces SCFA and exerts anti-tumor effects through the action of the intestinal microbiota (190). *Zhou* et al. evaluated the antitumor effects of *Tetrastigma hemsleyanum* polysaccharide (THP) using a lung tumor model. THP was able to increase the levels of secretory immunoglobulin A (SIgA) in the ileum and SCFAs in the cecum, and to improve the diversity of the intestinal microbial community, thereby restoring tumor-induced intestinal dysbiosis (191). *Tremella fuciformis* polysaccharides (TPs) are acidic heteropolysaccharides extracted from *Tremella fuciformis*. Berk. TPs exert a palliative effect on DSS-induced colitis by modulating tyrosine biosynthesis, tryptophan metabolism, and bile acid metabolism, while restoring the balance of the intestinal flora and the normal level of its microbial metabolites (192).

### 6.4 Flavonoids

Kaempferol is mainly derived from the ginger plant *Kaempferia galanga* L., which has received widespread attention for its anticancer and antioxidant properties (193). Kaempferol regulates intestinal flora by significantly increasing the abundance of beneficial bacteria and inhibiting the growth of potentially pathogenic bacteria, downregulating the secondary bile acid synthesis pathway, increasing the activity of G-protein-coupled receptors, and decreasing NOD-like receptor activity (194).

### 6.5 Terpenoids

Sterols maintain a diverse gut microbial environment and enrich the beneficial bacterium *Lactobacillus pentosus*. Significantly increased levels of SCFAs were found in fecal samples analyzed from mice treated with sterols. In addition, sterols reduced the transactivation effects of the PI3K/Akt signaling pathway, which regulated the expression levels of several apoptosis-related proteins, ultimately inducing apoptosis in colon cancer tumor cells (195).

### 6.6 Phenolic compounds

Inflammation is one of the most important factors in the development of intestinal tumors, and the close relationship between inflammation and tumors has made inflammation one of the important targets for anticancer therapy. Magnolol exhibits a significant anti-inflammatory effect on DSS-induced colitis, and the mechanism may be related to the restoration of tryptophan metabolites that inhibit colonic inflammation (196). Cranberries are rich in polyphenolic compounds, one of the most important being proanthocyanidins (C-PACs). Among other things, cranberry has been shown to attenuate animal diet-induced increases in secondary bile acids and decreases in SCFAs (197), suggesting that cranberry compounds have a positive effect on the regulation of intestinal microbial metabolites. C-PAC targeted the gut microbiome-esophageal metabolome axis to inhibit esophageal adenocarcinoma (EAC) progression by increasing the abundance of the beneficial bacteria *Lactococcus*, *Lactobacillus* and *Bifidobacterium*. C-PAC has been shown to reverse reflux-induced microbial dysregulation, attenuate bile acid metabolism and transport, and ultimately significantly inhibit EAC via the TLR/NF-κB/TP 53 signaling cascade (198).

### 6.7 Natural product extracts

Patchouli Essential Oil (PEO), derived from Pogostemon cablin (PC), and its derivatives, patchouli alcohol (PA) and pogostone (PO), stimulate SCFA producers in Apc^Min/+^ colon cancer mouse models and activate key SCFA-sensitive receptors (GPR 41, GPR 43 and GPR 109a). The gut microbiota of PEO-treated mice changed, with a significant reduction in the abundance of the *Bacteroidetes phylum* and a notable increase in the abundance of the *Firmicutes phylum (* 199). In addition, PA and PO significantly promoted the growth of a probiotic, eosinophilic Ackermannia, which has anti-inflammatory effects and protects the host intestinal mucosa (200).

### 6.8 Traditional Chinese medicine compound prescription

Phytopharmaceuticals have shown broad application prospects in the field of tumor therapy due to their multi-target and multi-link synergistic regulatory properties. Currently, the specific mechanism of this process has become a hot topic of research regarding how botanicals mediate tumor therapy by regulating the function of the gut flora and its microbial metabolites. (**Table 2**).

**Table 2:** Mechanisms of traditional Chinese medicine compounding for the treatment of tumors via intestinal microbial metabolites.

Chinese medicine compound prescription	Microbial metabolites	Mechanism	References
*Astragalus mongholicus* Bunge-*Curcuma aromatica* Salisb.	SCFAs	Increasing the content of SCFAs such as propionic acid and butyric acid in colon cancer cells, mediating the intestinal-derived SDF-1/CXCR 4 signaling pathway to repair the integrity of the intestinal barrier, decreasing the expression of Cyclin D1 and C-myc, and inhibiting tumor growth and metastasis.	(201)
Xianlian Jiedu Decoction	SCFAs	Reduced levels of inflammatory cytokines and decreased expression of β-conjugated proteins, COX-2, and iNOS proteins in colorectal tissues. Improves gut microbes and metabolic levels of associated SCFAs, sphingolipids and glycerophospholipids.	(202)
Shen-Bai-Jie-Du decoction	SCFAs	Increased production of SCFAs, activated G protein-coupled receptors, and inhibited HDAC. Decreased the proportion of M1-type macrophages, increased M2-type macrophages, down-regulated the expression levels of IL-1β, IL-6 pro-inflammatory factors, and up-regulated the expression levels of IL-10 anti-inflammatory factors.	(203)
PRM 1201	SCFAs	Regulates the composition of intestinal flora, increases the number of SCFA-producing bacteria and SCFA production, inhibits histone deacetylation and inhibits EMT in metastatic lesions thereby inhibiting CRC metastasis.	(204)
Huangqin Decoction	SCFAs	Improvement of intestinal dysbiosis, increase of *Clostridium* abundance and fecal butyric acid level, inhibition of microbial butyric acid-mediated PI3K/Akt pathway, induction of apoptosis, attenuation of intestinal inflammation, and reduction of tumor load in CRC.	(205)
Jianpi Yangzheng decoction	SCFAs	Remodeling the structure of intestinal flora, enhancing the production of SCFAs, inhibiting the recruitment of MDSCs and reducing the production of inflammatory inhibitory factors thereby inhibiting the formation of pre-metastatic microenvironment of gastric cancer.	(206)
Jianpi Jiedu decoction	Tryptophan metabolites (indole-3-acetaldehyde, 3-methylindole, 5-hydroxyindoleacetic acid, kynur­enine, 5-methoxyindoleacetate, and L-tryptophan.)	Altering the composition of the intestinal flora, increasing the abundance of beneficial intestinal bacteria, decreasing tryptophan metabolites, decreasing inflammation inhibiting the expression of AhR and M2-type tumor-associated macrophages, and enhancing anti-tumor immunity.	(207)
Wumei Wan	Tryptophan metabolites (3-indolepropionic acid)	Modulation of 3-indolepropionic acid in serum of CAC mice reduces the PI3K/Akt signaling pathway thereby inhibiting MDSCs	(208)
Pien-Tze-Huang	Bile acids (cholic acid, taurocholic acid)	Promotes the production of taurine and taurine, increases the content of bile acids and unsaturated fatty acids, and strengthens the function of the intestinal barrier. Inhibits PI 3 K-Akt, IL-17, TNF and cytokine-chemokine signaling	(209)
Xianglian Pill	Bile acids (DCA)	Reducing IL-6 and TNF-α expression and infiltration of pro-inflammatory macrophages, remodeling gut microbiota composition and bile acid metabolism to inhibit the TLR4/MyD 88 pathway, and thereby inhibiting colorectal cancer associated with a high-fat diet.	(211)
Sanhuang xiexin decoction	Bile acids	Reduced hepatocyte swelling in mice with colitis, inhibited IL-1β and TNF-α expression, and attenuated hepatic inflammation and cholestasis by regulating TLR4 NF-κB and bile acid metabolic pathways.	(212)
Qiang-Gan formula	Bile acids (Cholic Acid, Chenodeoxycholic Acid, DCA and LCA)	Decreased hepatic and serum bile acid concentrations, increased fecal lithobionic acid production, improved the structure of intestinal flora, promoted TGR5 expression, and inhibited NF-κB activation.	(213)
Siwu-Yin	Bile acids	Improvement of the intestinal flora in precancerous esophageal lesions and modulation of bile acid synthesis and secretion, thereby modulating macrophage polarization.	(214)

**Figure 3:**
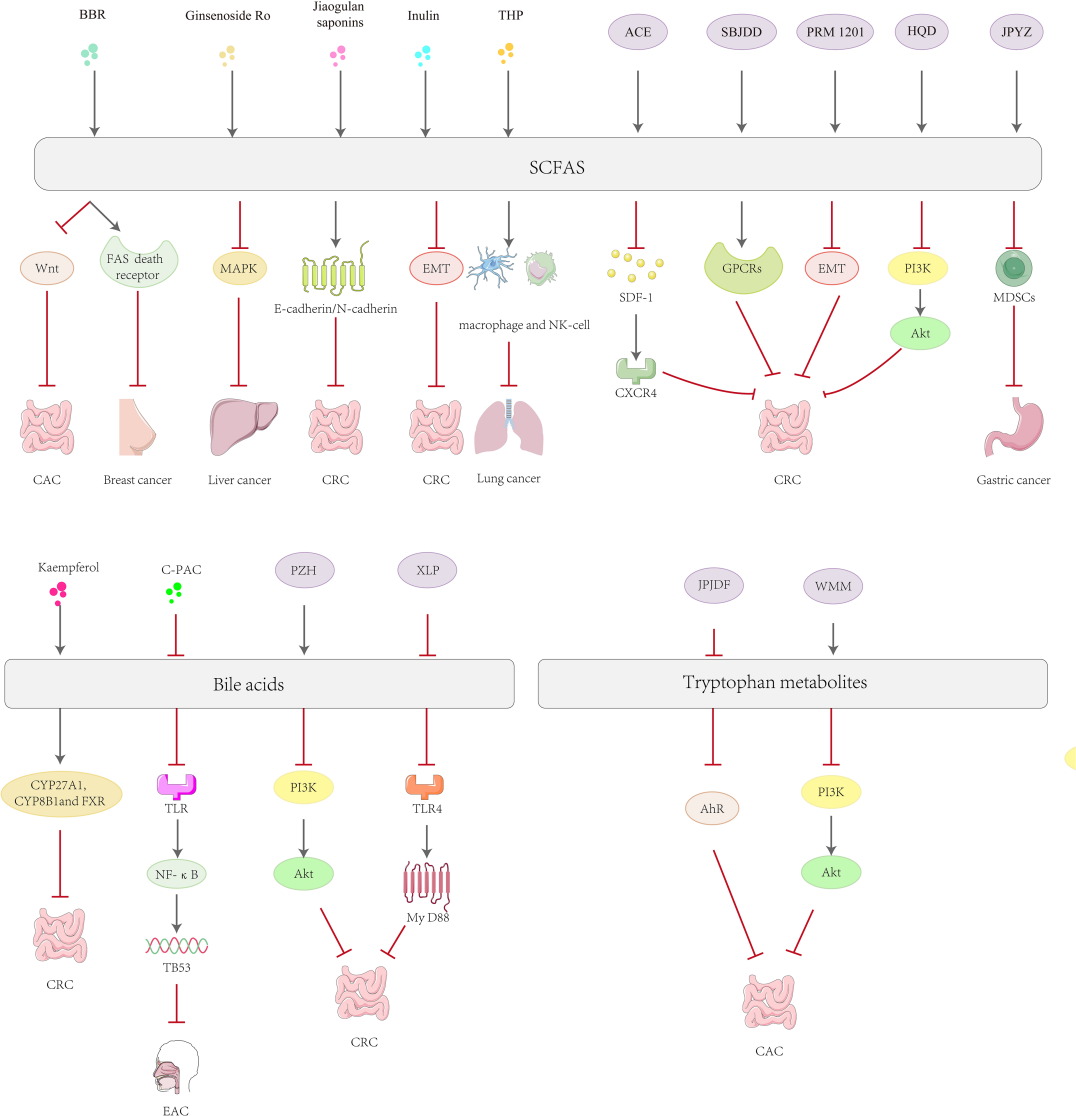
Mechanisms of natural product active ingredients and complexes in the treatment of tumors via gut microbial metabolites.

For example, some conventional drugs such as *Astragalus mongholicus* Bunge.-*Curcuma aromatica* Salisb. (ACE), which effectively delayed tumor progression in CT26 colon cancer-bearing mice, increased levels of SCFAs such as the gut microbial metabolites propionic acid and butyric acid in colon cancer cells and mediated the gut-derived stromal cell-derived factor-1 (SDF-1)/C-X-C chemokine receptor type 4 (CXCR 4) signaling pathway to inhibit tumor growth and metastasis (201). Xianlian Jiedu Decoction (XLJDD) treatment was effective in reducing the level of inflammatory response in a mouse model of colorectal cancer, the ability to reduce serum levels of inflammatory cytokines, and the ability to reduce levels of β-conjugated proteins, COX-2 and inducible nitric oxide synthase (iNOS) protein expression in colorectal tissue. Mechanistically, XLJDD improved gut dysbiosis and associated metabolic levels of SCFAs, sphingolipids and glycerophospholipids. It also increased the abundance of *Enterobacteriaceae* and *Zeylococcus-like probiotics*, as well as butyric and isovaleric acid levels (202). Shen-Bai-Jie-Du-Decoction (SBJDD) may promote the production of SCFAs by modulating the composition of intestinal flora, a process that further induces the polarization of M2-like macrophages, attenuates intestinal inflammation, restores intestinal barrier function and inhibits colorectal cancer cell proliferation (203). PRM 1201 inhibits CRC metastasis by regulating the abundance and metabolism of SCFA-producing bacteria, effectively suppressing histone deacetylation and inhibiting EMT in metastatic lesions (204). *Zhu* et al. used 16 S rRNA sequencing and gas chromatography-mass spectrometry (GC-MS) to detect changes in intestinal flora and fecal SCFAs after Huangqin Decoction (HQD) administration, respectively. The results showed that HQD improved intestinal flora dysbiosis, increased *Clostridium* abundance and fecal butyric acid levels, and inhibited the activity of PI3K/Akt pathway (205). Jianpi Yangzheng decoction (JPYZ) inhibits the formation of the pre-metastatic microenvironment of gastric cancer (GC) by remodeling the intestinal flora and increasing the production of its metabolite SCFA, which inhibits the infiltration of myeloid-derived suppressor cells (MDSC) and the production of inflammatory factors (206). Moreover, Jianpi Jiedu Decoction (JPJDF) inhibits the secretion of tryptophan metabolites, effectively reduces inflammation and significantly restores intestinal barrier function in colon cancer mice. It inhibited the expression of AhR and M2-type tumor associated macrophages polarization, thereby promoting tumor immunity and inhibiting the growth of colitis-associated colorectal cancer (CAC) caused by colonic inflammation (207). Wumei Wan(WMW) can restore the balance between pathogenic and probiotic bacteria in the intestinal tract. Early administration of WMW significantly regulated serum 3-indole propionic acid levels in mice with colon cancer and prevented colon cancer by inhibiting MDSCs by decreasing the PI3K/Akt signaling pathway (208). Pien-Tze-Huang (PZH) dose-dependently inhibited colorectal tumorigenesis in AOM/DSS-treated and Apc^min/+^ mice, promoted the production of beneficial metabolites such as taurine and hypotaurine, increased the levels of bile acids and unsaturated fatty acids, and significantly enhanced intestinal barrier function. Transcriptomic analysis further revealed that PZH inhibited signaling pathways such as PI3K-Akt, IL-17, TNF and cytokine-chemokines, effectively blocking oncogenic and pro-inflammatory pathways, which in turn inhibited colorectal carcinogenesis (209). Bile acids are mainly synthesized in the liver and secreted into the intestine, and the conversion of primary bile acids into secondary bile acids occurs with the help of the intestinal microbiota. Secondary bile acids are also able to be reabsorbed into the liver via the enterohepatic circulation and participate in the process of bile acid resynthesis and excretion (210). Certain secondary bile acids (e.g., DCA and lithocholic acid) are thought to have pro-cancer effects. They can alter gene expression by activating nuclear receptors, promote cell proliferation and survival, and may even induce cancer cell invasion and metastasis. Xianglian Pill (XLP) can be used for the treatment of gastrointestinal disorders of the damp-heat type, and *Ye* et al. assessed the protective effect of XLP using AOM and DSS-induced CRC models in mice exposed to high-fat diet. XLP significantly reduced a microbial-derived metabolite of bile acids called fecal DCA by restoring ecological dysbiosis in the gut microbiota, thereby inhibiting the TLR4/Myeloid Differentiation Primary Response Protein 88 (MyD88) pathway and reducing M1 macrophage infiltration (211). Sanhuang xiexin decoction (SXD) was able to modulate the gut-liver axis immunomodulation to ameliorate DSS-induced colitis secondary to hepatic injury, and attenuate hepatic inflammation and cholestasis by improving TLR4-NF-κB and bile acid metabolic pathways (212). Treatment of feed-induced methionine- and choline-deficient diet-induced nonalcoholic steatohepatitis (NASH) mice with Qiang-Gan formula (QGE) showed that QGE treatment altered the gut microbiota of NASH mice and led to an increase in fecal lithocholic acid content (213). In addition, the ability of Siwu-Yin (SWY) to improve the intestinal flora of rats with esophageal precancerous lesions is also related to the regulation of bile acid synthesis and secretion (214) (**Figure 3**).

Phytopharmaceuticals have demonstrated significant efficacy and benefits in the treatment of tumors, and their actions involve a variety of complex mechanisms. However, research into the role of phytopharmaceuticals in regulating microbial metabolites is still at a preliminary stage and more in-depth studies are needed to clarify their mechanism of action and precise efficacy.

## 7 Challenges and perspectives

The relationship between microbial metabolites and tumor immunotherapy is complex. Several studies have shown that microorganisms and their metabolites have a non-negligible impact on tumorigenesis and development, as well as on the efficacy and prognostic ability of tumor immunotherapy, and that their role includes regulation of immune cells and modulation of anti-inflammatory pathways. At the same time, several studies have shown that microbial metabolites not only affect the development of gastrointestinal tumors, but also influence the TME outside the gastrointestinal tract via the gut-X axis, which in turn affects tumor immunotherapy. Therefore, many researchers have also actively explored the use of the positive aspects of microbial metabolites in conjunction with tumor immunotherapy, including probiotic supplementation therapy, fecal microbiota transplantation methods etc., with the aim of increasing bacterial diversity, attenuating acute and long-term treatment-related toxicity, and achieving amazing results in improving immunotherapy response and prognosis, and individualizing treatment. However, we should also be aware of the increasing interest in the relationship between gut flora and anti-tumor immunotherapy, the essence of which is that the flora modulates the immune response through metabolic pathways, while the mechanisms of action between identified microbial metabolites and tumors are unclear, especially for tumors outside the gastrointestinal tract, leading to an inability to explain the differences in the number of species of gut microbial metabolites in different types of cancer. For example, studies have shown that butyrate in SCFA maintains gut integrity, preserves normal metabolic immune function and is beneficial for immunotherapeutic response, but a study showed that serum butyrate and juxtate concentrations did not significantly correlate with longer progression-free survival in French and Italian melanoma patients. Current studies of gut microbiota regulation of anti-tumor immunity have focused on the regulation of immune cells and inflammatory factors outside tumor cells, and there are no reports of gut microbes causing changes inside tumor cells to evade immune surveillance. On the other hand, most studies have focused on the relationship between the gut microbiota and cancer development, and metabolites have not been sufficiently studied; many microbial metabolites have not yet been identified. Meanwhile, the role of the gut microbiota in other immunotherapies, such as cytokine therapy and immunovaccine therapy, remains understudied. With further elucidation of the mechanism of influence of gut flora metabolites on tumor immune escape, targeted drug therapy and individualized anti-tumor therapy will be further developed, and gut flora modulation will become an effective and necessary adjuvant anti-tumor immunotherapy.

## References

[1] Topalian SL Drake CG Pardoll DM. Immune checkpoint blockade: a common denominator approach to cancer therapy. Cancer Cell. (2015) 27:450–61. doi: 10.1016/j.ccell.2015.03.001 PMC4400238 25858804

[2] Rooks MG Garrett WS. Gut microbiota metabolites and host immunity. Nature reviews. Immunology. (2016) 16:341–52. 10.1038/nri.2016.42 PMC5541232 27231050

[3] Zhou L Wei X. Ocular immune-related adverse events associated with immune checkpoint inhibitors in lung cancer. Front Immunol. (2021) 12:701951. doi: 10.3389/fimmu.2021.701951 34504488 PMC8421677

[4] Hanahan D. Hallmarks of cancer: new dimensions. . Cancer Discovery. (2022) 12:31–46. doi: 10.1158/2159-8290.CD-21-1059 35022204

[5] Arthur JC Perez-Chanona E Mühlbauer M Tomkovich S Uronis JM Fan TJ. Intestinal inflammation targets cancer-inducing activity of the microbiota. Science(New York N.Y.). (6103) 2012 338:120–3. 10.1126/science.1224820 PMC3645302 22903521

[6] Duan H Wang L Huangfu M Li H. The impact of microbiota-derived short-chain fatty acids on macrophage activities in disease: Mechanisms and therapeutic potentials. Biomedicine pharmacotherapy = Biomedecine pharmacotherapie. (2023) 165:115276. doi: 10.1016/j.biopha.2023.115276 37542852

[7] Levy M Thaiss CA Elinav E. Metabolites: messengers between the microbiota and the immune system. Genes Dev. (2016) 30:1589–97. doi: 10.1101/gad.284091.116 PMC4973288 27474437

[8] Sipos A Ujlaki G Mikó E Maka E Szabó J Uray K. The role of the microbiome in ovarian cancer: mechanistic insights into oncobiosis and to bacterial metabolite signaling. Mol medicine(Cambridge Mass.). (2021) 27:33. 10.1186/s10020-021-00295-2 PMC8017782 33794773

[9] Hezaveh K Shinde RS Klötgen A Halaby MJ Lamorte S Ciudad MT. Tryptophan-derived microbial metabolites activate the aryl hydrocarbon receptor in tumor-associated macrophages to suppress anti-tumor immunity. Immunity. (2022) 55:324–340.e8. doi: 10.1016/j.immuni.2022.01.006 35139353 PMC8888129

[10] Kiss B Mikó E Sebő É Toth J Ujlaki G Szabó J. Oncobiosis and microbial metabolite signaling in pancreatic adenocarcinoma. Cancers. (2020) 12. doi: 10.3390/cancers12051068 PMC7281526 32344895

[11] Bhagat TD Von Ahrens D Dawlaty M Zou Y Baddour J Achreja A. Lactate-mediated epigenetic reprogramming regulates formation of human pancreatic cancer-associated fibroblasts. eLife. (2019) 8. doi: 10.7554/eLife.50663 PMC6874475 31663852

[12] Mullins TD Kern HF Metzgar RS. Ultrastructural differentiation of sodium butyrate-treated human pancreatic adenocarcinoma cell lines. Pancreas. (1991) 6:578–87. doi: 10.1097/00006676-199109000-00012 1946315

[13] Chen AN Luo Y Yang YH Fu JT Geng XM Shi JP. Lactylation a novel metabolic reprogramming code: current status and prospects. Front Immunol. (2021) 12:688910. doi: 10.3389/fimmu.2021.688910 34177945 PMC8222712

[14] Rees DO Crick PJ Jenkins GJ Wang Y Griffiths WJ Brown TH. Comparison of the composition of bile acids in bile of patients with adenocarcinoma of the pancreas and benign disease. J Steroid Biochem Mol Biol. (2017) 174:290–5. doi: 10.1016/j.jsbmb.2017.10.011 PMC5668629 29031685

[15] Plewa S Horała A Dereziński P Klupczynska A Nowak-Markwitz E Matysiak J. Usefulness of amino acid profiling in ovarian cancer screening with special emphasis on their role in cancerogenesis. Int J Mol Sci. (2017) 18. doi: 10.3390/ijms18122727 PMC5751328 29258187

[16] Hilvo M de Santiago I Gopalacharyulu P Schmitt WD Budczies J Kuhberg M. Accumulated metabolites of hydroxybutyric acid serve as diagnostic and prognostic biomarkers of ovarian high-grade serous carcinomas. Cancer Res. (2016) 76:796–804. doi: 10.1158/0008-5472.CAN-15-2298 26685161 PMC4762194

[17] Zhou M Guan W Walker LD Mezencev R Benigno BB Gray A. Rapid mass spectrometric metabolic profiling of blood sera detects ovarian cancer with high accuracy. Cancer Epidemiol Biomarkers Prev Publ Am Assoc Cancer Res cosponsored by Am Soc Prev Oncol. (2010) 19:2262–71. doi: 10.1158/1055-9965.EPI-10-0126 20699376

[18] Ke C Hou Y Zhang H Fan L Ge T Guo B. Large-scale profiling of metabolic dysregulation in ovarian cancer. Int J Cancer. (2015) 136:516–26. doi: 10.1002/ijc.v136.3 24895217

[19] Yang Q Wang B Zheng Q Li H Meng X Zhou F. A review of gut microbiota-derived metabolites in tumor progression and cancer therapy. Advanced science(Weinheim Baden-Wurttemberg Germany). (2023) 10:e2207366. doi: 10.1002/advs.202207366 36951547 PMC10214247

[20] Aghamajidi A Maleki Vareki S. The effect of the gut microbiota on systemic and anti-tumor immunity and response to systemic therapy against cancer. Cancers. (2022) 14. doi: 10.3390/cancers14153563 PMC9330582 35892821

[21] Li Y Jiang H Wang X Liu X Huang Y Wang Z. Crosstalk between the gut and brain: importance of the fecal microbiota in patient with brain tumors. Front Cell infection Microbiol. (2022) 12:881071. doi: 10.3389/fcimb.2022.881071 PMC9247299 35782130

[22] Tremaroli V Bäckhed F. Functional interactions between the gut microbiota and host metabolism. Nature. (7415) 2012 489:242–9. 10.1038/nature11552 22972297

[23] González-Sánchez P DeNicola GM. The microbiome(s) and cancer: know thy neighbor(s). J Pathol. (2021) 254:332–43. doi: 10.1002/path.v254.4 33723873

[24] Oliphant K Allen-Vercoe E. Macronutrient metabolism by the human gut microbiome: major fermentation by-products and their impact on host health. Microbiome. (2019) 7:91. doi: 10.1186/s40168-019-0704-8 31196177 PMC6567490

[25] den Besten G Lange K Havinga R van Dijk TH Gerding A van Eunen K. Gut-derived short-chain fatty acids are vividly assimilated into host carbohydrates and lipids. American journal of physiology. Gastrointestinal liver Physiol. (2013) 305:G900–10. doi: 10.1152/ajpgi.00265.2013 24136789

[26] Wang G Huang S Wang Y Cai S Yu H Liu H. Bridging intestinal immunity and gut microbiota by metabolites. Cell Mol Life Sci CMLS. (2019) 76:3917–37. doi: 10.1007/s00018-019-03190-6 PMC6785585 31250035

[27] Wong CC Yu J. Gut microbiota in colorectal cancer development and therapy. Nature reviews. Clin Oncol. (2023) 20:429–52. 10.1038/s41571-023-00766-x 37169888

[28] Cummings JH Pomare EW Branch WJ Naylor CP Macfarlane GT. Short chain fatty acids in human large intestine portal hepatic and venous blood. Gut. (1987) 28:1221–7. doi: 10.1136/gut.28.10.1221 PMC1433442 3678950

[29] Topping DL Clifton PM. Short-chain fatty acids and human colonic function: roles of resistant starch and nonstarch polysaccharides. Physiol Rev. (2001) 81:1031–64. doi: 10.1152/physrev.2001.81.3.1031 11427691

[30] Kelly CJ Zheng L Campbell EL Saeedi B Scholz CC Bayless AJ. Crosstalk between microbiota-derived short-chain fatty acids and intestinal epithelial HIF augments tissue barrier function. Cell Host Microbe. (2015) 17:662–71. doi: 10.1016/j.chom.2015.03.005 PMC4433427 25865369

[31] Lewis K Lutgendorff F Phan V Söderholm JD Sherman PM McKay DM. Enhanced translocation of bacteria across metabolically stressed epithelia is reduced by butyrate. Inflammatory bowel Dis. (2010) 16:1138–48. doi: 10.1002/ibd.21177 20024905

[32] Singh N Gurav A Sivaprakasam S Brady E Padia R Shi H. Activation of Gpr109a receptor for niacin and the commensal metabolite butyrate suppresses colonic inflammation and carcinogenesis. Immunity. (2014) 40:128–39. doi: 10.1016/j.immuni.2013.12.007 PMC4305274 24412617

[33] Ben Q Sun Y Chai R Qian A Xu B Yuan Y. Dietary fiber intake reduces risk for colorectal adenoma: a meta-analysis. Gastroenterology. (2014) 146:689–699.e6. doi: 10.1053/j.gastro.2013.11.003 24216326

[34] Sipe LM Chaib M Pingili AK Pierre JF Makowski L. Microbiome bile acids and obesity: How microbially modified metabolites shape anti-tumor immunity. Immunol Rev. (2020) 295:220–39. doi: 10.1111/imr.v295.1 PMC7841960 32320071

[35] Shen J Yang L You K Chen T Su Z Cui Z. Indole-3-acetic acid alters intestinal microbiota and alleviates ankylosing spondylitis in mice. Front Immunol. (2022) 13:762580. doi: 10.3389/fimmu.2022.762580 35185872 PMC8854167

[36] Xue C Li G Zheng Q Gu X Shi Q Su Y. Tryptophan metabolism in health and disease. Cell Metab. (2023) 35:1304–26. doi: 10.1016/j.cmet.2023.06.004 37352864

[37] Sun M Ma N He T Johnston LJ Ma X. Tryptophan(Trp) modulates gut homeostasis via aryl hydrocarbon receptor(AhR). Crit Rev Food Sci Nutr. (2020) 60:1760–8. doi: 10.1080/10408398.2019.1598334 30924357

[38] Wei GZ Martin KA Xing PY Agrawal R Whiley L Wood TK. Tryptophan-metabolizing gut microbes regulate adult neurogenesis via the aryl hydrocarbon receptor. Proc Natl Acad Sci United States America. (2021) 118. doi: 10.1073/pnas.2021091118 PMC8271728 34210797

[39] Bhattarai Y Jie S Linden DR Ghatak S Mars RAT Williams BB. Bacterially derived tryptamine increases mucus release by activating a host receptor in a mouse model of inflammatory bowel disease. iScience. (2020) 23:101798. doi: 10.1016/j.isci.2020.101798 33299969 PMC7702010

[40] Cho CE Taesuwan S Malysheva OV Bender E Tulchinsky NF Yan J. Trimethylamine-N-oxide(TMAO) response to animal source foods varies among healthy young men and is influenced by their gut microbiota composition: A randomized controlled trial. Mol Nutr Food Res. (2017) 61. doi: 10.1002/mnfr.201600324 27377678

[41] Roager HM Licht TR. Microbial tryptophan catabolites in health and disease. Nat Commun. (2018) 9:3294. doi: 10.1038/s41467-018-05470-4 30120222 PMC6098093

[42] Sanchez-Azofra A Gu W Masso-Silva JA Sanz-Rubio D Marin-Oto M Cubero P. Inflammation biomarkers in OSA chronic obstructive pulmonary disease and chronic obstructive pulmonary disease/OSA overlap syndrome. J Clin sleep Med JCSM Off Publ Am Acad Sleep Med. (2023) 19:1447–56. doi: 10.5664/jcsm.10600 PMC10394367 37082823

[43] Fan Y Pedersen O. Gut microbiota in human metabolic health and disease. Nature reviews. Microbiology. (2021) 19:55–71. 32887946 10.1038/s41579-020-0433-9

[44] Zhang Z Zhang H Chen T Shi L Wang D Tang D. Regulatory role of short-chain fatty acids in inflammatory bowel disease. Cell communication Signaling CCS. (2022) 20:64. doi: 10.1186/s12964-022-00869-5 35546404 PMC9097439

[45] Wilson RAM Evans TRJ Fraser AR Nibbs RJB. Immune checkpoint inhibitors: new strategies to checkmate cancer. Clin Exp Immunol. (2018) 191:133–48. doi: 10.1111/cei.13081 PMC5758374 29139554

[46] Igney FH Krammer PH. Immune escape of tumors: apoptosis resistance and tumor counterattack. J leukocyte Biol. (2002) 71:907–20. doi: 10.1189/jlb.71.6.907 12050175

[47] Vinay DS Ryan EP Pawelec G Talib WH Stagg J Elkord E. Immune evasion in cancer: Mechanistic basis and therapeutic strategies. Semin Cancer Biol. (2015) 35 Suppl S185-s198. doi: 10.1016/j.semcancer.2015.03.004 25818339

[48] Bouzin C Brouet A De Vriese J Dewever J Feron O. Effects of vascular endothelial growth factor on the lymphocyte-endothelium interactions: identification of caveolin-1 and nitric oxide as control points of endothelial cell anergy. J immunology(Baltimore Md. 1950). (2007) 178:1505–11. doi: 10.4049/jimmunol.178.3.1505 17237399

[49] Oleinika K Nibbs RJ Graham GJ Fraser AR. Suppression subversion and escape: the role of regulatory T cells in cancer progression. Clin Exp Immunol. (2013) 171:36–45. doi: 10.1111/j.1365-2249.2012.04657.x 23199321 PMC3530093

[50] Krummel MF Allison JP. CD28 and CTLA-4 have opposing effects on the response of T cells to stimulation. J Exp Med. (1995) 182:459–65. doi: 10.1084/jem.182.2.459 PMC2192127 7543139

[51] Alegre ML Frauwirth KA. T-cell regulation by CD28 and CTLA-4. Nat Rev Immunol. (2001) 1:220–8. doi: 10.1038/35105024 11905831

[52] Leach DR Krummel MF Allison JP. Enhancement of antitumor immunity by CTLA-4 blockade. Science(New York N.Y.). (5256) 1996 271:1734–6. 10.1126/science.271.5256.1734 8596936

[53] Dagar G Gupta A Masoodi T Nisar S Merhi M Hashem S. Harnessing the potential of CAR-T cell therapy: progress challenges and future directions in hematological and solid tumor treatments. J Trans Med. (2023) 21:449. doi: 10.1186/s12967-023-04292-3 PMC10327392 37420216

[54] Cheng W Kang K Zhao A Wu Y. Dual blockade immunotherapy targeting PD-1/PD-L1 and CTLA-4 in lung cancer. J Hematol Oncol. (2024) 17:54. doi: 10.1186/s13045-024-01581-2 39068460 PMC11283714

[55] Ishida Y Agata Y Shibahara K Honjo T. Induced expression of PD-1 a novel member of the immunoglobulin gene superfamily upon programmed cell death. EMBO J. (1992) 11:3887–95. doi: 10.1002/j.1460-2075.1992.tb05481.x PMC556898 1396582

[56] Ortega MA Boaru DL De-Leon-Oliva D Fraile-Martinez O García-Montero C Rios L. PD-1/PD-L1 axis: implications in immune regulation cancer progression and translational applications. J Mol medicine(Berlin Germany). (2024) 102:987–1000. doi: 10.1007/s00109-024-02463-3 38935130

[57] Keir ME Butte MJ Freeman GJ Sharpe AH. PD-1 and its ligands in tolerance and immunity. Annu Rev Immunol. (2008) 26:677–704. doi: 10.1146/annurev.immunol.26.021607.090331 18173375 PMC10637733

[58] Latchman Y Wood CR Chernova T Chaudhary D Borde M Chernova I. PD-L2 is a second ligand for PD-1 and inhibits T cell activation. Nat Immunol. (2001) 2:261–8. doi: 10.1038/85330 11224527

[59] Hui E Cheung J Zhu J Su X Taylor MJ Wallweber HA. T cell costimulatory receptor CD28 is a primary target for PD-1-mediated inhibition. Science(New York N.Y.). (6332) 2017 355:1428–33. 10.1126/science.aaf1292 PMC6286077 28280247

[60] Thompson RH Gillett MD Cheville JC Lohse CM Dong H Webster WS. Costimulatory B7-H1 in renal cell carcinoma patients: Indicator of tumor aggressiveness and potential therapeutic target. Proc Natl Acad Sci United States America. (2004) 101:17174–9. doi: 10.1073/pnas.0406351101 PMC534606 15569934

[61] Curiel TJ Wei S Dong H Alvarez X Cheng P Mottram P. Blockade of B7-H1 improves myeloid dendritic cell-mediated antitumor immunity. Nat Med. (2003) 9:562–7. doi: 10.1038/nm863 12704383

[62] Hodi FS O'Day SJ McDermott DF Weber RW Sosman JA Haanen JB. Improved survival with ipilimumab in patients with metastatic melanoma. New Engl J Med. (2010) 363:711–23. doi: 10.1056/NEJMoa1003466 PMC3549297 20525992

[63] Inoue Y Inui N. Associations between immune-related adverse events and prognosis in cancer patients receiving immune checkpoint inhibitor therapy. Internal medicine(Tokyo Japan). (2024). doi: 10.2169/internalmedicine.4654-24 PMC12854964 39566979

[64] Shimoyama K Nakajima A Minari Y. The first report of bickerstaff brainstem encephalitis induced by atezolizumab for metastatic breast cancer. Acta Med Okayama. (2024) 78:407–12. 10.18926/AMO/67665 39467659

[65] Dana H Chalbatani GM Jalali SA Mirzaei HR Grupp SA Suarez ER. CAR-T cells: Early successes in blood cancer and challenges in solid tumors. A. cta Pharm Sinica. B. (2021) 11:1129–47. 10.1016/j.apsb.2020.10.020 PMC8144892 34094824

[66] Bird RE Hardman KD Jacobson JW Johnson S Kaufman BM Lee SM. Single-chain antigen-binding proteins. Science(New York N.Y.). (4877) 1988 242:423–6. 10.1126/science.3140379 3140379

[67] Zmievskaya E Valiullina A Ganeeva I Petukhov A Rizvanov A Bulatov E. Application of CAR-T cell therapy beyond oncology: autoimmune diseases and viral infections. Biomedicines. (2021) 9. doi: 10.3390/biomedicines9010059 PMC7827151 33435454

[68] Huston JS Levinson D Mudgett-Hunter M Tai MS Novotný J Margolies MN. Protein engineering of antibody binding sites: recovery of specific activity in an anti-digoxin single-chain Fv analogue produced in Escherichia coli. Proc Natl Acad Sci United States America. (1988) 85:5879–83. doi: 10.1073/pnas.85.16.5879 PMC281868 3045807

[69] Gupta A Dagar G Rehmani MU Prasad CP Saini D Singh M. CAR T-cell therapy in cancer: Integrating nursing perspectives for enhanced patient care. Asia-Pacific J Oncol Nurs. (2024) 11:100579. doi: 10.1016/j.apjon.2024.100579 PMC11417177 39315365

[70] Ferment B Arnulf B. CAR-T cells immunotherapy in multiple myeloma: Present and future. Bull du Cancer. (2021) 108:S65–s72. 10.1016/j.bulcan.2021.09.005 34920809

[71] Locke FL Neelapu SS Bartlett NL Lekakis LJ Jacobson CA Braunschweig I. Tocilizumab prophylaxis following axicabtagene ciloleucel in relapsed or refractory large B-cell lymphoma. Transplant Cell Ther. (2024) 30:1065–79. doi: 10.1016/j.jtct.2024.08.018 39187161

[72] Neelapu SS Chavez JC Sehgal AR Epperla N Ulrickson M Bachy E. Three-year follow-up analysis of axicabtagene ciloleucel in relapsed/refractory indolent non-Hodgkin lymphoma(ZUMA-5). Blood. (2024) 143:496–506. doi: 10.1182/blood.2023021243 37879047 PMC10934297

[73] Wang M Siddiqi T Gordon LI Kamdar M Lunning M Hirayama AV. Lisocabtagene maraleucel in relapsed/refractory mantle cell lymphoma: primary analysis of the mantle cell lymphoma cohort from TRANSCEND NHL 001 a phase I multicenter seamless design study. J Clin Oncol Off J Am Soc Clin Oncol. (2024) 42:1146–57. doi: 10.1200/JCO.23.02214 PMC11741176 38072625

[74] Cohen AD. CAR T-cell therapy against B-cell maturation antigen in multiple myeloma. Clin Adv Hematol Oncol H&O. (2018) 16:804–6. 30843888

[75] Majzner RG Mackall CL. Tumor antigen escape from CAR T-cell therapy. Cancer Discovery. (2018) 8:1219–26. doi: 10.1158/2159-8290.CD-18-0442 30135176

[76] Maude SL Teachey DT Porter DL Grupp SA. CD19-targeted chimeric antigen receptor T-cell therapy for acute lymphoblastic leukemia. Blood. (2015) 125:4017–23. doi: 10.1182/blood-2014-12-580068 PMC4481592 25999455

[77] Kim JM Chen DS. Immune escape to PD-L1/PD-1 blockade: seven steps to success(or failure). Ann Oncol Off J Eur Soc Med Oncol. (2016) 27:1492–504. doi: 10.1093/annonc/mdw217 27207108

[78] Hollingsworth RE Jansen K. Turning the corner on therapeutic cancer vaccines. NPJ Vaccines. (2019) 4:7. doi: 10.1038/s41541-019-0103-y 30774998 PMC6368616

[79] Kantoff PW Higano CS Shore ND Berger ER Small EJ Penson DF. Sipuleucel-T immunotherapy for castration-resistant prostate cancer. New Engl J Med. (2010) 363:411–22. doi: 10.1056/NEJMoa1001294 20818862

[80] Tiwari A Alcover K Carpenter E Thomas K Krum J Nissen A. Utility of cell-based vaccines as cancer therapy: Systematic review and meta-analysis. Hum Vaccines immunotherapeutics. (2024) 20:2323256. doi: 10.1080/21645515.2024.2323256 PMC10984131 38544385

[81] Xi HB Wang GX Fu B Liu WP Li Y. Survivin and PSMA loaded dendritic cell vaccine for the treatment of prostate cancer. Biol Pharm Bull. (2015) 38:827–35. doi: 10.1248/bpb.b14-00518 25787895

[82] Han CL Yan YC Yan LJ Meng GX Yang CC Liu H. Efficacy and security of tumor vaccines for hepatocellular carcinoma: a systemic review and meta-analysis of the last 2 decades. J Cancer Res Clin Oncol. (2023) 149:1425–41. doi: 10.1007/s00432-022-04008-y PMC11797259 35482077

[83] Yamanaka R Homma J Yajima N Tsuchiya N Sano M Kobayashi T. Clinical evaluation of dendritic cell vaccination for patients with recurrent glioma: results of a clinical phase I/II trial. Clin Cancer Res an Off J Am Assoc Cancer Res. (2005) 11:4160–7. doi: 10.1158/1078-0432.CCR-05-0120 15930352

[84] Agus A Clément K Sokol H. Gut microbiota-derived metabolites as central regulators in metabolic disorders. Gut. (2021) 70:1174–82. doi: 10.1136/gutjnl-2020-323071 PMC8108286 33272977

[85] Chen L Zhernakova DV Kurilshikov A Andreu-Sánchez S Wang D Augustijn HE. Influence of the microbiome diet and genetics on inter-individual variation in the human plasma metabolome. Nat Med. (2022) 28:2333–43. doi: 10.1038/s41591-022-02014-8 PMC9671809 36216932

[86] Burcelin R Serino M Chabo C Garidou L Pomié C Courtney M. Metagenome and metabolism: the tissue microbiota hypothesis. Diabetes Obes Metab. (2013) 15 Suppl 3:61–70. 24003922 10.1111/dom.12157

[87] Wikoff WR Anfora AT Liu J Schultz PG Lesley SA Peters EC. Metabolomics analysis reveals large effects of gut microflora on mammalian blood metabolites. Proc Natl Acad Sci United States America. (2009) 106:3698–703. doi: 10.1073/pnas.0812874106 PMC2656143 19234110

[88] Dumas ME. The microbial-mammalian metabolic axis: beyond simple metabolism. Cell Metab. (2011) 13:489–90. doi: 10.1016/j.cmet.2011.04.005 21531329

[89] Puertollano E Kolida S Yaqoob P. Biological significance of short-chain fatty acid metabolism by the intestinal microbiome. Curr Opin Clin Nutr Metab Care. (2014) 17:139–44. doi: 10.1097/MCO.0000000000000025 24389673

[90] Mikó E Kovács T Sebő É Tóth J Csonka T Ujlaki G. Microbiome-microbial metabolome-cancer cell interactions in breast cancer-familiar but unexplored. Cells. (2019) 8. 10.3390/cells8040293 PMC6523810 30934972

[91] Davar D Dzutsev AK McCulloch JA Rodrigues RR Chauvin JM Morrison RM. Fecal microbiota transplant overcomes resistance to anti-PD-1 therapy in melanoma patients. Science(New York N.Y.). (6529) 2021 371:595–602. 10.1126/science.abf3363 PMC8097968 33542131

[92] Sivan A Corrales L Hubert N Williams JB Aquino-Michaels K Earley ZM. Commensal Bifidobacterium promotes antitumor immunity and facilitates anti-PD-L1 efficacy. Science(New York N.Y.). (6264) 2015 350:1084–9. 10.1126/science.aac4255 PMC4873287 26541606

[93] Smith M Dai A Ghilardi G Amelsberg KV Devlin SM Pajarillo R. Gut microbiome correlates of response and toxicity following anti-CD19 CAR T cell therapy. Nat Med. (2022) 28:713–23. doi: 10.1038/s41591-022-01702-9 PMC9434490 35288695

[94] Zhou CB Zhou YL Fang JY. Gut microbiota in cancer immune response and immunotherapy. Trends Cancer. (2021) 7:647–60. doi: 10.1016/j.trecan.2021.01.010 33674230

[95] Tsvetikova SA Koshel EI. Microbiota and cancer: host cellular mechanisms activated by gut microbial metabolites. Int J Med Microbiol IJMM. (2020) 310:151425. doi: 10.1016/j.ijmm.2020.151425 32423739

[96] Qin N Yang F Li A Prifti E Chen Y Shao L. Alterations of the human gut microbiome in liver cirrhosis. Nature. (7516) 2014 513:59–64. 10.1038/nature13568 25079328

[97] Iida N Mizukoshi E Yamashita T Yutani M Seishima J Wang Z. Chronic liver disease enables gut Enterococcus faecalis colonization to promote liver carcinogenesis. Nat Cancer. (2021) 2:1039–54. doi: 10.1038/s43018-021-00251-3 35121877

[98] Ponziani FR Bhoori S Castelli C Putignani L Rivoltini L Del Chierico F. Hepatocellular carcinoma is associated with gut microbiota profile and inflammation in nonalcoholic fatty liver disease. Hepatology(Baltimore Md.). (2019) 69:107–20. 10.1002/hep.30036 29665135

[99] Ma C Han M Heinrich B Fu Q Zhang Q Sandhu M. Gut microbiome-mediated bile acid metabolism regulates liver cancer via NKT cells. Science(New York N.Y.). (2018) 360. doi: 10.1126/science.aan5931 PMC6407885 29798856

[100] Yoshimoto S Loo TM Atarashi K Kanda H Sato S Oyadomari S. Obesity-induced gut microbial metabolite promotes liver cancer through senescence secretome. Nature. (7456) 2013 499:97–101. 10.1038/nature12347 23803760

[101] Yamagishi R Kamachi F Nakamura M Yamazaki S Kamiya T Takasugi M. Gasdermin D-mediated release of IL-33 from senescent hepatic stellate cells promotes obesity-associated hepatocellular carcinoma. Sci Immunol. (2022) 7:eabl7209. doi: 10.1126/sciimmunol.abl7209 35749514

[102] Han K Nam J Xu J Sun X Huang X Animasahun O. Generation of systemic antitumour immunity via the in situ modulation of the gut microbiome by an orally administered inulin gel. Nat Biomed Eng. (2021) 5:1377–88. doi: 10.1038/s41551-021-00749-2 PMC8595497 34168321

[103] Lee PC Wu CJ Hung YW Lee CJ Chi CT Lee IC. Gut microbiota and metabolites associate with outcomes of immune checkpoint inhibitor-treated unresectable hepatocellular carcinoma. J immunotherapy Cancer. (2022) 10. doi: 10.1136/jitc-2022-004779 PMC9226985 35738801

[104] Han S Bao X Zou Y Wang L Li Y Yang L. d-lactate modulates M2 tumor-associated macrophages and remodels immunosuppressive tumor microenvironment for hepatocellular carcinoma. Sci Adv. (2023) 9:eadg2697. doi: 10.1126/sciadv.adg2697 37467325 PMC10355835

[105] Zheng Y Wang T Tu X Huang Y Zhang H Tan D. Gut microbiome affects the response to anti-PD-1 immunotherapy in patients with hepatocellular carcinoma. J immunotherapy Cancer. (2019) 7:193. doi: 10.1186/s40425-019-0650-9 PMC6651993 31337439

[106] Elkrief A El Raichani L Richard C Messaoudene M Belkaid W Malo J. Antibiotics are associated with decreased progression-free survival of advanced melanoma patients treated with immune checkpoint inhibitors. Oncoimmunology. (2019) 8:e1568812. doi: 10.1080/2162402X.2019.1568812 30906663 PMC6422373

[107] Vétizou M Pitt JM Daillère R Lepage P Waldschmitt N Flament C. Anticancer immunotherapy by CTLA-4 blockade relies on the gut microbiota. Science(New York N.Y.). (6264) 2015 350:1079–84. 10.1126/science.aad1329 PMC4721659 26541610

[108] Routy B Le Chatelier E Derosa L Duong CPM Alou MT Daillère R. Gut microbiome influences efficacy of PD-1-based immunotherapy against epithelial tumors. Science(New York N.Y.). (6371) 2018 359:91–7. 10.1126/science.aan3706 29097494

[109] Su X Gao Y Yang R. Gut microbiota-derived tryptophan metabolites maintain gut and systemic homeostasis. Cells. (2022) 11. doi: 10.3390/cells11152296 PMC9330295 35892593

[110] Flores R Shi J Fuhrman B Xu X Veenstra TD Gail MH. Fecal microbial determinants of fecal and systemic estrogens and estrogen metabolites: a cross-sectional study. J Trans Med. (2012) 10:253. doi: 10.1186/1479-5876-10-253 PMC3552825 23259758

[111] Liu L Fu Q Li T Shao K Zhu X Cong Y. Gut microbiota and butyrate contribute to nonalcoholic fatty liver disease in premenopause due to estrogen deficiency. PloS One. (2022) 17:e0262855. doi: 10.1371/journal.pone.0262855 35108315 PMC8809533

[112] Wang MY Sang LX Sun SY. Gut microbiota and female health. World J Gastroenterol. (2024) 30:1655–62. doi: 10.3748/wjg.v30.i12.1655 PMC11008377 38617735

[113] Hosseini E Grootaert C Verstraete W Van de Wiele T. Propionate as a health-promoting microbial metabolite in the human gut. Nutr Rev. (2011) 69:245–58. doi: 10.1111/j.1753-4887.2011.00388.x 21521227

[114] Wu Z Pfeiffer RM Byrd DA Wan Y Ansong D Clegg-Lamptey JN. Associations of circulating estrogens and estrogen metabolites with fecal and oral microbiome in postmenopausal women in the Ghana breast health study. Microbiol Spectr. (2023) 11:e0157223. doi: 10.1128/spectrum.01572-23 37341612 PMC10433996

[115] Goedert JJ Jones G Hua X Xu X Yu G Flores R. Investigation of the association between the fecal microbiota and breast cancer in postmenopausal women: a population-based case-control pilot study. J Natl Cancer Institute. (2015) 107. 10.1093/jnci/djv147 PMC4554191 26032724

[116] Goedert JJ Hua X Bielecka A Okayasu I Milne GL Jones GS. Postmenopausal breast cancer and oestrogen associations with the IgA-coated and IgA-noncoated faecal microbiota. Br J Cancer. (2018) 118:471–9. doi: 10.1038/bjc.2017.435 PMC5830593 29360814

[117] Luu TH Michel C Bard JM Dravet F Nazih H Bobin-Dubigeon C. Intestinal proportion of blautia sp. is associated with clinical stage and histoprognostic grade in patients with early-stage breast cancer. Nutr Cancer. (2017) 69:267–75. doi: 10.1080/01635581.2017.1263750 28094541

[118] Yin Y Sichler A Ecker J Laschinger M Liebisch G Höring M. Gut microbiota promote liver regeneration through hepatic membrane phospholipid biosynthesis. J Hepatol. (2023) 78:820–35. doi: 10.1016/j.jhep.2022.12.028 36681162

[119] Mikó E Vida A Kovács T Ujlaki G Trencsényi G Márton J. Lithocholic acid a bacterial metabolite reduces breast cancer cell proliferation and aggressiveness. Biochimica et biophysica acta. Bioenergetics. (2018) 1859:958–74. 10.1016/j.bbabio.2018.04.002 29655782

[120] Di Modica M Gargari G Regondi V Bonizzi A Arioli S Belmonte B. Gut microbiota condition the therapeutic efficacy of trastuzumab in HER2-positive breast cancer. Cancer Res. (2021) 81:2195–206. doi: 10.1158/0008-5472.CAN-20-1659 33483370

[121] Li D Yu S Long Y Shi A Deng J Ma Y. Tryptophan metabolism: Mechanism-oriented therapy for neurological and psychiatric disorders. Front Immunol. (2022) 13:985378. doi: 10.3389/fimmu.2022.985378 36159806 PMC9496178

[122] Rothhammer V Mascanfroni ID Bunse L Takenaka MC Kenison JE Mayo L. Type I interferons and microbial metabolites of tryptophan modulate astrocyte activity and central nervous system inflammation via the aryl hydrocarbon receptor. Nat Med. (2016) 22:586–97. doi: 10.1038/nm.4106 PMC4899206 27158906

[123] Chyan YJ Poeggeler B Omar RA Chain DG Frangione B Ghiso J. Potent neuroprotective properties against the Alzheimer beta-amyloid by an endogenous melatonin-related indole structure indole-3-propionic acid. J Biol Chem. (1999) 274:21937–42. doi: 10.1074/jbc.274.31.21937 10419516

[124] Gao J Xu K Liu H Liu G Bai M Peng C. Impact of the gut microbiota on intestinal immunity mediated by tryptophan metabolism. Front Cell infection Microbiol. (2018) 8:13. doi: 10.3389/fcimb.2018.00013 PMC5808205 29468141

[125] Blackmer-Raynolds LD Sampson TR. The gut-brain axis goes viral. Cell Host Microbe. (2022) 30:283–5. doi: 10.1016/j.chom.2022.02.013 35271800

[126] Wang M Zhang L Chang W Zhang Y. The crosstalk between the gut microbiota and tumor immunity: Implications for cancer progression and treatment outcomes. Front Immunol. (2022) 13:1096551. doi: 10.3389/fimmu.2022.1096551 36726985 PMC9885097

[127] Green GBH Cox-Holmes AN Potier ACE Marlow GH McFarland BC. Modulation of the immune environment in glioblastoma by the gut microbiota. Biomedicines. (2024) 12. doi: 10.3390/biomedicines12112429 PMC11591702 39594997

[128] Cong J Liu P Han Z Ying W Li C Yang Y. Bile acids modified by the intestinal microbiota promote colorectal cancer growth by suppressing CD8(+) T cell effector functions. Immunity. (2024) 57:876–889.e11. doi: 10.1016/j.immuni.2024.02.014 38479384

[129] Pant K Venugopal SK Lorenzo Pisarello MJ Gradilone SA. The role of gut microbiome-derived short-chain fatty acid butyrate in hepatobiliary diseases. Am J Pathol. (2023) 193:1455–67. doi: 10.1016/j.ajpath.2023.06.007 PMC10548274 37422149

[130] Mager LF Burkhard R Pett N Cooke NCA Brown K Ramay H. Microbiome-derived inosine modulates response to checkpoint inhibitor immunotherapy. Science(New York N.Y.). (6510) 2020 369:1481–9. 10.1126/science.abc3421 32792462

[131] Liu X Lu B Tang H Jia X Zhou Q Zeng Y. Gut microbiome metabolites molecular mimicry and species-level variation drive long-term efficacy and adverse event outcomes in lung cancer survivors. EBioMedicine. (2024) 109:105427. doi: 10.1016/j.ebiom.2024.105427 39471749 PMC11550776

[132] Botticelli A Vernocchi P Marini F Quagliariello A Cerbelli B Reddel S. Gut metabolomics profiling of non-small cell lung cancer(NSCLC) patients under immunotherapy treatment. J Trans Med. (2020) 18:49. doi: 10.1186/s12967-020-02231-0 PMC6998840 32014010

[133] Song B Zhao K Zhou S Xue Y Lu H Jia X. Association of the gut microbiome with fecal short-chain fatty acids lipopolysaccharides and obesity in young Chinese college students. Front Nutr. (2023) 10:1057759. doi: 10.3389/fnut.2023.1057759 37139436 PMC10150786

[134] Luu M Weigand K Wedi F Breidenbend C Leister H Pautz S. Regulation of the effector function of CD8(+) T cells by gut microbiota-derived metabolite butyrate. Sci Rep. (2018) 8:14430. doi: 10.1038/s41598-018-32860-x 30258117 PMC6158259

[135] Mirji G Worth A Bhat SA El Sayed M Kannan T Goldman AR. The microbiome-derived metabolite TMAO drives immune activation and boosts responses to immune checkpoint blockade in pancreatic cancer. Sci Immunol. (2022) 7:eabn0704. doi: 10.1126/sciimmunol.abn0704 36083892 PMC9925043

[136] Armstrong D Cameron RG. Comparison of liver cancer and nodules induced in rats by deoxycholic acid diet with or without prior initiation. Cancer Lett. (1991) 57:153–7. doi: 10.1016/0304-3835(91)90209-Z 1673872

[137] Režen T Rozman D Kovács T Kovács P Sipos A Bai P. The role of bile acids in carcinogenesis. Cell Mol Life Sci CMLS. (2022) 79:243. doi: 10.1007/s00018-022-04278-2 35429253 PMC9013344

[138] Coutzac C Jouniaux JM Paci A Schmidt J Mallardo D Seck A. Systemic short chain fatty acids limit antitumor effect of CTLA-4 blockade in hosts with cancer. Nat Commun. (2020) 11:2168. doi: 10.1038/s41467-020-16079-x 32358520 PMC7195489

[139] Zhu J Luo L Tian L Yin S Ma X Cheng S. Aryl hydrocarbon receptor promotes IL-10 expression in inflammatory macrophages through src-STAT3 signaling pathway. Front Immunol. (2018) 9:2033. doi: 10.3389/fimmu.2018.02033 30283437 PMC6156150

[140] Chng SH Kundu P Dominguez-Brauer C Teo WL Kawajiri K Fujii-Kuriyama Y. Ablating the aryl hydrocarbon receptor(AhR) in CD11c+ cells perturbs intestinal epithelium development and intestinal immunity. Sci Rep. (2016) 6:23820. doi: 10.1038/srep23820 27068235 PMC4828637

[141] Frankel TL Pasca di Magliano M. Immune sensing of microbial metabolites: Action at the tumor. Immunity. (2022) 55:192–4. doi: 10.1016/j.immuni.2022.01.009 35139348

[142] Ji Y Gao Y Chen H Yin Y Zhang W. Indole-3-acetic acid alleviates nonalcoholic fatty liver disease in mice via attenuation of hepatic lipogenesis and oxidative and inflammatory stress. Nutrients. (2019) 11. doi: 10.3390/nu11092062 PMC6769627 31484323

[143] Krishnan S Ding Y Saedi N Choi M Sridharan GV Sherr DH. Gut microbiota-derived tryptophan metabolites modulate inflammatory response in hepatocytes and macrophages. Cell Rep. (2018) 23:1099–111. doi: 10.1016/j.celrep.2018.03.109 PMC6392449 29694888

[144] Beaumont M Neyrinck AM Olivares M Rodriguez J de Rocca Serra A Roumain M. The gut microbiota metabolite indole alleviates liver inflammation in mice. FASEB J Off Publ Fed Am Societies Exp Biol. (2018) 32:fj201800544. doi: 10.1096/fj.201800544 PMC6219839 29906245

[145] Bansal T Alaniz RC Wood TK Jayaraman A. The bacterial signal indole increases epithelial-cell tight-junction resistance and attenuates indicators of inflammation. Proc Natl Acad Sci United States America. (2010) 107:228–33. doi: 10.1073/pnas.0906112107 PMC2806735 19966295

[146] Walter K Grosskopf H Karkossa I von Bergen M Schubert K. Proteomic characterization of the cellular effects of ahR activation by microbial tryptophan catabolites in endotoxin-activated human macrophages. Int J Environ Res Public Health. (2021) 18. doi: 10.3390/ijerph181910336 PMC8507890 34639632

[147] Mantovani A Sozzani S Locati M Allavena P Sica A. Macrophage polarization: tumor-associated macrophages as a paradigm for polarized M2 mononuclear phagocytes. Trends Immunol. (2002) 23:549–55. doi: 10.1016/S1471-4906(02)02302-5 12401408

[148] Ji Y Yin W Liang Y Sun L Yin Y Zhang W. Anti-inflammatory and anti-oxidative activity of indole-3-acetic acid involves induction of HO-1 and neutralization of free radicals in RAW264.7 cells. . Int J Mol Sci. (2020) 21. doi: 10.3390/ijms21051579 PMC7084870 32106625

[149] Ishihara Y Kado SY Hoeper C Harel S Vogel CFA. Role of NF-kB relB in aryl hydrocarbon receptor-mediated ligand specific effects. Int J Mol Sci. (2019) 20. doi: 10.3390/ijms20112652 PMC6600526 31151139

[150] Agista AZ Tanuseputero SA Koseki T Budijanto S Sultana H Ohsaki Y. Tryptamine a microbial metabolite in fermented rice bran suppressed lipopolysaccharide-induced inflammation in a murine macrophage model. Int J Mol Sci. (2022) 23. doi: 10.3390/ijms231911209 PMC9570467 36232510

[151] Liu YJ Tang B Wang FC Tang L Lei YY Luo Y. Parthenolide ameliorates colon inflammation through regulating Treg/Th17 balance in a gut microbiota-dependent manner. Theranostics. (2020) 10:5225–41. doi: 10.7150/thno.43716 PMC7196297 32373209

[152] Chanmee T Ontong P Konno K Itano N. Tumor-associated macrophages as major players in the tumor microenvironment. Cancers. (2014) 6:1670–90. doi: 10.3390/cancers6031670 PMC4190561 25125485

[153] Buchta Rosean C Bostic RR Ferey JCM Feng TY Azar FN Tung KS. Preexisting commensal dysbiosis is a host-intrinsic regulator of tissue inflammation and tumor cell dissemination in hormone receptor-positive breast cancer. Cancer Res. (2019) 79:3662–75. doi: 10.1158/0008-5472.CAN-18-3464 PMC6983951 31064848

[154] Li R Zhou R Wang H Li W Pan M Yao X. Gut microbiota-stimulated cathepsin K secretion mediates TLR4-dependent M2 macrophage polarization and promotes tumor metastasis in colorectal cancer. Cell Death differentiation. (2019) 26:2447–63. doi: 10.1038/s41418-019-0312-y PMC6889446 30850734

[155] Liu T Guo Z Song X Liu L Dong W Wang S. High-fat diet-induced dysbiosis mediates MCP-1/CCR2 axis-dependent M2 macrophage polarization and promotes intestinal adenoma-adenocarcinoma sequence. J Cell Mol Med. (2020) 24:2648–62. doi: 10.1111/jcmm.14984 PMC7028862 31957197

[156] Busbee PB Menzel L Alrafas HR Dopkins N Becker W Miranda K. Indole-3-carbinol prevents colitis and associated microbial dysbiosis in an IL-22-dependent manner. JCI Insight. (2020) 5. doi: 10.1172/jci.insight.127551 PMC7030851 31941837

[157] Zhu C Xie Q Zhao B. The role of AhR in autoimmune regulation and its potential as a therapeutic target against CD4 T cell mediated inflammatory disorder. Int J Mol Sci. (2014) 15:10116–35. doi: 10.3390/ijms150610116 PMC4100143 24905409

[158] Safa M Tavasoli B Manafi R Kiani F Kashiri M Ebrahimi S. Indole-3-carbinol suppresses NF-κB activity and stimulates the p53 pathway in pre-B acute lymphoblastic leukemia cells. Tumour Biol J Int Soc Oncodevelopmental Biol Med. (2015) 36:3919–30. doi: 10.1007/s13277-014-3035-1 25589462

[159] Yan XJ Qi M Telusma G Yancopoulos S Madaio M Satoh M. Indole-3-carbinol improves survival in lupus-prone mice by inducing tandem B- and T-cell differentiation blockades. Clin immunology(Orlando Fla.). (2009) 131:481–94. 10.1016/j.clim.2009.01.013 19278904

[160] Pierre S Chevallier A Teixeira-Clerc F Ambolet-Camoit A Bui LC Bats AS. Aryl hydrocarbon receptor-dependent induction of liver fibrosis by dioxin. Toxicological Sci an Off J Soc Toxicol. (2014) 137:114–24. doi: 10.1093/toxsci/kft236 24154488

[161] Weng JR Tsai CH Kulp SK Chen CS. Indole-3-carbinol as a chemopreventive and anti-cancer agent. Cancer Lett. (2008) 262:153–63. doi: 10.1016/j.canlet.2008.01.033 PMC2814317 18314259

[162] Hung SC Kuo KL Huang HL Lin CC Tsai TH Wang CH. Indoxyl sulfate suppresses endothelial progenitor cell-mediated neovascularization. Kidney Int. (2016) 89:574–85. doi: 10.1016/j.kint.2015.11.020 26880454

[163] Zeb F Naqeeb H Osaili T Faris ME Ismail LC Obaid RS. Molecular crosstalk between polyphenols and gut microbiota in cancer prevention. Nutr research(New York N.Y.). (2024) 124:21–42. 10.1016/j.nutres.2024.01.012 38364552

[164] Mitchem JB Brennan DJ Knolhoff BL Belt BA Zhu Y Sanford DE. Targeting tumor-infiltrating macrophages decreases tumor-initiating cells relieves immunosuppression and improves chemotherapeutic responses. Cancer Res. (2013) 73:1128–41. doi: 10.1158/0008-5472.CAN-12-2731 PMC3563931 23221383

[165] DeNardo DG Brennan DJ Rexhepaj E Ruffell B Shiao SL Madden SF. Leukocyte complexity predicts breast cancer survival and functionally regulates response to chemotherapy. Cancer Discovery. (2011) 1:54–67. doi: 10.1158/2159-8274.CD-10-0028 22039576 PMC3203524

[166] Qu R Zhang Y Ma Y Zhou X Sun L Jiang C. Role of the gut microbiota and its metabolites in tumorigenesis or development of colorectal cancer. Advanced science(Weinheim Baden-Wurttemberg Germany). (2023) 10:e2205563. doi: 10.1002/advs.202205563 37263983 PMC10427379

[167] Jaye K Chang D Li CG Bhuyan DJ. Gut metabolites and breast cancer: the continuum of dysbiosis breast cancer risk and potential breast cancer therapy. Int J Mol Sci. (2022) 23. doi: 10.3390/ijms23169490 PMC9409206 36012771

[168] Létourneau S Krieg C Pantaleo G Boyman O. IL-2- and CD25-dependent immunoregulatory mechanisms in the homeostasis of T-cell subsets. J Allergy Clin Immunol. (2009) 123:758–62. 10.1016/j.jaci.2009.02.011 19348914

[169] Cibrián D Sánchez-Madrid F. CD69: from activation marker to metabolic gatekeeper. Eur J Immunol. (2017) 47:946–53. doi: 10.1002/eji.201646837 PMC6485631 28475283

[170] Hwang YJ Yun MO Jeong KT Park JH. Uremic toxin indoxyl 3-sulfate regulates the differentiation of Th2 but not of Th1 cells to lessen allergic asthma. Toxicol Lett. (2014) 225:130–8. doi: 10.1016/j.toxlet.2013.11.027 24291743

[171] Kamal A Rao MP Swapna P Srinivasulu V Bagul C Shaik AB. Synthesis of β-carboline-benzimidazole conjugates using lanthanum nitrate as a catalyst and their biological evaluation. Organic biomolecular Chem. (2014) 12:2370–87. doi: 10.1039/C3OB42236D 24604306

[172] Nyström S Govender M Yap SH Kamarulzaman A Rajasuriar R Larsson M. HIV-infected individuals on ART with impaired immune recovery have altered plasma metabolite profiles. Open Forum Infect Dis. (2021) 8:ofab288. doi: 10.1093/ofid/ofab288 34258318 PMC8271132

[173] Borghi M Pariano M Solito V Puccetti M Bellet MM Stincardini C. Targeting the aryl hydrocarbon receptor with indole-3-aldehyde protects from vulvovaginal candidiasis via the IL-22-IL-18 cross-talk. Front Immunol. (2019) 10:2364. doi: 10.3389/fimmu.2019.02364 31681274 PMC6798081

[174] Ghosh S Das SK Sinha K Ghosh B Sen K Ghosh N. The emerging role of natural products in cancer treatment. Arch Toxicol. (2024) 98:2353–91. doi: 10.1007/s00204-024-03786-3 38795134

[175] Luo Y Yin S Lu J Zhou S Shao Y Bao X. Tumor microenvironment: a prospective target of natural alkaloids for cancer treatment. Cancer Cell Int. (2021) 21:386. doi: 10.1186/s12935-021-02085-6 34284780 PMC8290600

[176] Sun X Zhang Y Cheng G Zhu T Zhang Z Xiong L. Berberine improves DSS-induced colitis in mice by modulating the fecal-bacteria-related bile acid metabolism. Biomedicine pharmacotherapy = Biomedecine pharmacotherapie. (2023) 167:115430. doi: 10.1016/j.biopha.2023.115430 37683590

[177] Eaden J Abrams K Ekbom A Jackson E Mayberry J. Colorectal cancer prevention in ulcerative colitis: a case-control study. Alimentary Pharmacol Ther. (2000) 14:145–53. doi: 10.1046/j.1365-2036.2000.00698.x 10651654

[178] Wang M Ma Y Yu G Zeng B Yang W Huang C. Integration of microbiome metabolomics and transcriptome for in-depth understanding of berberine attenuates AOM/DSS-induced colitis-associated colorectal cancer. Biomedicine pharmacotherapy = Biomedecine pharmacotherapie. (2024) 179:117292. doi: 10.1016/j.biopha.2024.117292 39151314

[179] Ma W Zhang Y Yu M Wang B Xu S Zhang J. *In-vitro* and *in-vivo* anti-breast cancer activity of synergistic effect of berberine and exercise through promoting the apoptosis and immunomodulatory effects. Int Immunopharmacol. (2020) 87:106787. doi: 10.1016/j.intimp.2020.106787 32707493

[180] Mao N Yu Y Lu X Yang Y Liu Z Wang D. Preventive effects of matrine on LPS-induced inflammation in RAW 264.7 cells and intestinal damage in mice through the TLR4/NF-κB/MAPK pathway. Int Immunopharmacol. (2024) 143:113432. doi: 10.1016/j.intimp.2024.113432 39447411

[181] Zhang Y Hao R Chen J Li S Huang K Cao H. Health benefits of saponins and its mechanisms: perspectives from absorption metabolism and interaction with gut. Crit Rev Food Sci Nutr. (2024) 64:9311–32. doi: 10.1080/10408398.2023.2212063 37216483

[182] Li Z Song Y Xu W Chen J Zhou R Yang M. Pulsatilla chinensis saponins improve SCFAs regulating GPR43-NLRP3 signaling pathway in the treatment of ulcerative colitis. J ethnopharmacology. (2023) 308:116215. doi: 10.1016/j.jep.2023.116215 36806339

[183] Ai Z Liu S Zhang J Hu Y Tang P Cui L. Ginseng glucosyl oleanolate from ginsenoside ro exhibited anti-liver cancer activities via MAPKs and gut microbiota *in vitro*/vivo. J Agric Food Chem. (2024) 72:7845–60. doi: 10.1021/acs.jafc.3c08150 38501913

[184] Xie J Luo M Chen Q Zhang Q Qin L Wang Y. Hypolipidemic effect and gut microbiota regulation of Gypenoside aglycones in rats fed a high-fat diet. J ethnopharmacology. (2024) 328:118066. doi: 10.1016/j.jep.2024.118066 38499259

[185] Wu H Lai W Wang Q Zhou Q Zhang R Zhao Y. Gypenoside induces apoptosis by inhibiting the PI3K/AKT/mTOR pathway and enhances T-cell antitumor immunity by inhibiting PD-L1 in gastric cancer. Front Pharmacol. (2024) 15:1243353. doi: 10.3389/fphar.2024.1243353 38482051 PMC10933075

[186] Khan I Huang G Li XA Liao W Leong WK Xia W. Mushroom polysaccharides and jiaogulan saponins exert cancer preventive effects by shaping the gut microbiota and microenvironment in Apc(Min/+) mice. Pharmacol Res. (2019) 148:104448. doi: 10.1016/j.phrs.2019.104448 31499195

[187] Li H Dong T Tao M Zhao H Lan T Yan S. Fucoidan enhances the anti-tumor effect of anti-PD-1 immunotherapy by regulating gut microbiota. Food Funct. (2024) 15:3463–78. doi: 10.1039/D3FO04807A 38456333

[188] Miller KD O'Connor S Pniewski KA Kannan T Acosta R Mirji G. Acetate acts as a metabolic immunomodulator by bolstering T-cell effector function and potentiating antitumor immunity in breast cancer. Nat Cancer. (2023) 4:1491–507. doi: 10.1038/s43018-023-00636-6 PMC10615731 37723305

[189] Shoaib M Shehzad A Omar M Rakha A Raza H Sharif HR. Inulin: Properties health benefits and food applications. Carbohydr polymers. (2016) 147:444–54. doi: 10.1016/j.carbpol.2016.04.020 27178951

[190] Wang C Lan T Chen Z Wang X Han Y Yang N. The preventive effects of inulin cellulose and their mixture on colorectal cancer liver metastasis in mice by regulating gut microbiota. J Food Sci. (2023) 88:4705–17. doi: 10.1111/1750-3841.16772 37815692

[191] Zhou F Lu Y Sun T Sun L Wang B Lu J. Antitumor effects of polysaccharides from Tetrastigma hemsleyanum Diels et Gilg via regulation of intestinal flora and enhancing immunomodulatory effects *in vivo* . Front Immunol. (2022) 13:1009530. doi: 10.3389/fimmu.2022.1009530 36389762 PMC9650377

[192] Xu Y Xie L Zhang Z Zhang W Tang J He X. Tremella fuciformis Polysaccharides Inhibited Colonic Inflammation in Dextran Sulfate Sodium-Treated Mice via Foxp3+ T Cells Gut Microbiota and Bacterial Metabolites. Front Immunol. (2021) 12:648162. doi: 10.3389/fimmu.2021.648162 33868283 PMC8049506

[193] Auyeung KK Han QB Ko JK. Astragalus membranaceus: A Review of its Protection Against Inflammation and Gastrointestinal Cancers. Am J Chin Med. (2016) 44:1–22. doi: 10.1142/S0192415X16500014 26916911

[194] Li X Khan I Huang G Lu Y Wang L Liu Y. Kaempferol acts on bile acid signaling and gut microbiota to attenuate the tumor burden in ApcMin/+ mice. Eur J Pharmacol. (2022) 918:174773. doi: 10.1016/j.ejphar.2022.174773 35065044

[195] Ma H Yu Y Wang M Li Z Xu H Tian C. Correlation between microbes and colorectal cancer: tumor apoptosis is induced by sitosterols through promoting gut microbiota to produce short-chain fatty acids. Apoptosis an Int J programmed Cell Death. (2019) 24:168–83. doi: 10.1007/s10495-018-1500-9 30506375

[196] Zhao L Xiao HT Mu HX Huang T Lin ZS Zhong LLD. Magnolol a natural polyphenol attenuates dextran sulfate sodium-induced colitis in mice. Molecules(Basel Switzerland). (2017) 22. doi: 10.3390/molecules22071218 PMC6152296 28726741

[197] Rodríguez-Morató J Matthan NR Liu J de la Torre R Chen CO. Cranberries attenuate animal-based diet-induced changes in microbiota composition and functionality: a randomized crossover controlled feeding trial. J Nutr Biochem. (2018) 62:76–86. doi: 10.1016/j.jnutbio.2018.08.019 30269035

[198] Weh KM Howard CL Zhang Y Tripp BA Clarke JL Howell AB. Prebiotic proanthocyanidins inhibit bile reflux-induced esophageal adenocarcinoma through reshaping the gut microbiome and esophageal metabolome. JCI Insight. (2024) 9. doi: 10.1172/jci.insight.168112 PMC11063939 38329812

[199] Leong W Huang G Liao W Xia W Li X Su Z. Traditional Patchouli essential oil modulates the host's immune responses and gut microbiota and exhibits potent anti-cancer effects in Apc(Min /+) mice. . Pharmacol Res. (2022) 176:106082. doi: 10.1016/j.phrs.2022.106082 35032662

[200] Gu J Sun R Wang Q Liu F Tang D Chang X. Standardized astragalus mongholicus bunge-curcuma aromatica salisb. Extract efficiently suppresses colon cancer progression through gut microbiota modification in CT26-bearing mice. Front Pharmacol. (2021) 12:714322. doi: 10.3389/fphar.2021.714322 34531745 PMC8438123

[201] de Vos WM. Microbe Profile: Akkermansia muciniphila: a conserved intestinal symbiont that acts as the gatekeeper of our mucosa. Microbiology(Reading England). (2017) 163:646–8. doi: 10.1099/mic.0.000444 28530168

[202] Zong S Ye H Ye Z He Y Zhang X Ye M. Polysaccharides from Lachnum sp. Inhibited colitis-associated colon tumorigenesis in mice by modulating fecal microbiota and metabolites. Int Immunopharmacol. (2022) 108:108656. doi: 10.1016/j.intimp.2022.108656 35390743

[203] Huang M Zhang Y Ni M Shen M Tao Y Shen W. Shen-Bai-Jie-Du decoction suppresses the progression of colorectal adenoma to carcinoma through regulating gut microbiota and short-chain fatty acids. . Chin Med. (2024) 19:149. doi: 10.1186/s13020-024-01019-4 39465423 PMC11514841

[204] Jia R Shao S Zhang P Yuan Y Rong W An Z. PRM1201 effectively inhibits colorectal cancer metastasis via shaping gut microbiota and short- chain fatty acids. Phytomedicine Int J phytotherapy phytopharmacology. (2024) 132:155795. doi: 10.1016/j.phymed.2024.155795 38878524

[205] Zhu JJ Liu HY Yang LJ Fang Z Fu R Chen JB. Anti-tumour effect of Huangqin Decoction on colorectal cancer mice through microbial butyrate mediated PI3K/Akt pathway suppression. J Med Microbiol. (2023) 72. doi: 10.1099/jmm.0.001692 37195736

[206] Zhu X Zhang X Shen J Zheng S Li H Han B. Gut microbiota-dependent modulation of pre-metastatic niches by Jianpi Yangzheng decoction in the prevention of lung metastasis of gastric cancer. Phytomedicine Int J phytotherapy phytopharmacology. (2024) 128:155413. doi: 10.1016/j.phymed.2024.155413 38513377

[207] Chang Y Ou Q Zhou X Nie K Zheng P Liu J. Jianpi Jiedu decoction suppresses colorectal cancer growth by inhibiting M2 polarization of TAMs through the tryptophan metabolism-AhR pathway. Int Immunopharmacol. (2024) 138:112610. doi: 10.1016/j.intimp.2024.112610 38963982

[208] Lu ZH Ding Y Wang YJ Chen C Yao XR Yuan XM. Early administration of Wumei Wan inhibit myeloid-derived suppressor cells via PI3K/Akt pathway and amino acids metabolism to prevent colitis-associated colorectal cancer. J ethnopharmacology. (2024) 333:118260. doi: 10.1016/j.jep.2024.118260 38685367

[209] Gou H Su H Liu D Wong CC Shang H Fang Y. Traditional medicine pien tze huang suppresses colorectal tumorigenesis through restoring gut microbiota and metabolites. Gastroenterology. (2023) 165:1404–19. doi: 10.1053/j.gastro.2023.08.052 37704113

[210] Pushpass RG Alzoufairi S Jackson KG Lovegrove JA. Circulating bile acids as a link between the gut microbiota and cardiovascular health: impact of prebiotics probiotics and polyphenol-rich foods. Nutr Res Rev. (2022) 35:161–80. doi: 10.1017/S0954422421000081 33926590

[211] Ye C Wu C Li Y Chen C Li X Zhang J. Traditional medicine Xianglian pill suppresses high-fat diet-related colorectal cancer via inactivating TLR4/MyD88 by remodeling gut microbiota composition and bile acid metabolism. J ethnopharmacology. (2024) 333:118411. doi: 10.1016/j.jep.2024.118411 38824980

[212] Li L Wang Y Zhao L Ye G Shi F Li Y. Sanhuang xiexin decoction ameliorates secondary liver injury in DSS-induced colitis involve regulating inflammation and bile acid metabolism. J ethnopharmacology. (2022) 299:115682. doi: 10.1016/j.jep.2022.115682 36058478

[213] Li Q Li M Li F Zhou W Dang Y Zhang L. Qiang-Gan formula extract improves non-alcoholic steatohepatitis via regulating bile acid metabolism and gut microbiota in mice. J ethnopharmacology. (2020) 258:112896. doi: 10.1016/j.jep.2020.112896 32325178

[214] Shi HJ Chen XY Chen XR Wu ZB Li JY Sun YQ. Chinese medicine formula siwu-yin inhibits esophageal precancerous lesions by improving intestinal flora and macrophage polarization. Front Pharmacol. (2022) 13:812386. doi: 10.3389/fphar.2022.812386 35308250 PMC8927885

